# *In vitro* adaptation and characterization of attenuated hypervariable region 1 swap chimeras of hepatitis C virus

**DOI:** 10.1371/journal.ppat.1009720

**Published:** 2021-07-19

**Authors:** Christina Holmboe Olesen, Elias H. Augestad, Fulvia Troise, Jens Bukh, Jannick Prentoe

**Affiliations:** 1 Copenhagen Hepatitis C Program (CO-HEP), Department of Infectious Diseases, Hvidovre Hospital, Hvidovre, Denmark; 2 Copenhagen Hepatitis C Program (CO-HEP), Department of Immunology and Microbiology, Faculty of Health and Medical Sciences, University of Copenhagen, Copenhagen, Denmark; 3 Ceinge Biotecnologie Avanzate Via Gaetano Salvatore, Napoli, Italy; Princeton University, UNITED STATES

## Abstract

Hepatitis C virus (HCV) chronically infects 70 million people worldwide with an estimated annual disease-related mortality of 400,000. A vaccine could prevent spread of this pervasive human pathogen, but has proven difficult to develop, partly due to neutralizing antibody evasion mechanisms that are inherent features of the virus envelope glycoproteins, E1 and E2. A central actor is the E2 motif, hypervariable region 1 (HVR1), which protects several non-overlapping neutralization epitopes through an incompletely understood mechanism. Here, we show that introducing different HVR1-isolate sequences into cell-culture infectious JFH1-based H77 (genotype 1a) and J4 (genotype 1b) Core-NS2 recombinants can lead to severe viral attenuation. Culture adaptation of attenuated HVR1-swapped recombinants permitted us to identify E1/E2 substitutions at conserved positions both within and outside HVR1 that increased the infectivity of attenuated HVR1-swapped recombinants but were not adaptive for original recombinants. H77 recombinants with HVR1 from multiple other isolates consistently acquired substitutions at position 348 in E1 and position 385 in HVR1 of E2. Interestingly, HVR1-swapped J4 recombinants primarily acquired other substitutions: F291I (E1), F438V (E2), F447L/V/I (E2) and V710L (E2), indicating a different adaptation pathway. For H77 recombinants, the adaptive E1/E2 substitutions increased sensitivity to the neutralizing monoclonal antibodies AR3A and AR4A, whereas for J4 recombinants, they increased sensitivity to AR3A, while having no effect on sensitivity to AR4A. To evaluate effects of the substitutions on AR3A and AR4A binding, we performed ELISAs on extracted E1/E2 protein and performed immunoprecipitation of relevant viruses. However, extracted E1/E2 protein and immunoprecipitation of HCV particles only reproduced the neutralization phenotypes of the J4 recombinants. Finally, we found that the HVR1-swap E1/E2 substitutions decrease virus entry dependency on co-receptor SR-BI. Our study identifies E1/E2 positions that could be critical for intra-complex HVR1 interactions while emphasizing the need for developing novel tools for molecular studies of E1/E2 interactions.

## Author summary

Hepatitis C virus (HCV) employs several mechanisms to avoid eradication by the host immune system. This includes the virus’ ability to evade the B-cell response by occluding conserved neutralizing antibody (NAb) epitopes on the HCV envelope protein 1 (E1) and 2 (E2). An important component of HCV NAb evasion is the E2 motif, hypervariable region 1 (HVR1), which broadly regulates HCV neutralization sensitivity. As HVR1 displays a high level of structural flexibility, it has not been possible to infer HVR1 localization from solved structures of E2. Consequently, little is known about its interaction with the remaining part of the envelope proteins. In this study, we describe substitutions at conserved E1 and E2 positions both within and outside of HVR1 that appear critical for HVR1-specific E1/E2 function. We showed that these substitutions regulated protection of conserved neutralization epitopes and influenced receptor dependency of viral entry. Finally, testing the effect of these substitutions on NAb binding to extracted protein and in HCV immunoprecipitation we demonstrated the challenges of studying these phenomena outside the context of neutralization assays of infectious virus particles. Overall, this study contributes to our understanding of HVR1-mediated NAb protection which must be overcome in HCV vaccine strategies.

## Introduction

Globally, more than 70 million people are chronically infected with hepatitis C virus (HCV), and have an increased risk of developing liver cirrhosis and hepatocellular carcinoma. This is the cause of approximately 400,000 annual deaths, making HCV a global health concern [1]. Since 2011, direct-acting antivirals against HCV have greatly improved infection cure rates [2], but it is unlikely that global virus elimination can be achieved through treatment efforts alone. This is due to high drug cost and limited access to treatment, frequent undiagnosed infections, as well as emerging drug resistance and the risk of reinfection following treatment [3]. Thus, it seems clear that worldwide control of HCV will require a vaccine [4]. Vaccine development has proven difficult, partly because much remains unknown about the mechanisms of HCV evasion from neutralizing antibodies (NAbs) [4, 5].

HCV is an enveloped, positive-stranded RNA virus within the Flaviviridae family with six clinically relevant genotypes and multiple subtypes [6]. Embedded in the viral envelope are HCV glycoproteins E1 and E2. These form the E1/E2 heterodimer, which serves critical roles in virus entry and consequently is the main target of NAbs. The E2 sequence is highly diverse, particularly within the 26–27 N-terminal amino acids, termed hypervariable region 1 (HVR1) [7]. HVR1 plays an essential role in viral evasion from NAbs and may accumulate mutations to fine-tune NAb sensitivity [5, 8, 9]. Several studies have demonstrated that HVR1 protects HCV from NAbs targeting non-HVR1 epitopes [10–12], and it was recently shown that polymorphisms within HVR1 positions 400–404 regulate this protection through a mechanism involving perturbation of global conformation dynamics of the HCV envelope glycoproteins [8]. HVR1-mediated NAb protection has also been verified *in vivo*, suggesting that HVR1 function could directly impact infection outcome [13]. However, intra-protein interactions underlying this epitope protection have been difficult to study as the complete structure of the E1/E2 complex is still missing. Furthermore, due to its apparent structural flexibility, full-length HVR1 has been excluded from solved structures of monomeric E2 [14–17]. Thus, little is known of interactions between HVR1 and other parts of the E1/E2 heterodimer.

HCV is dependent on several cellular factors for entry into the host cell, including the co-receptors CD81 [18, 19] and scavenger receptor class B, type I (SR-BI) [20]. Interestingly, the removal of HVR1 increases the propensity of HCV to interact with CD81 and data suggests that SR-BI entry dependency of HCV is affected either directly by the deletion of HVR1 or by HVR1 deletion fitness compensatory envelope protein mutations [12, 21, 22]. Furthermore, HCV recombinants with increased occlusion of conserved neutralization epitopes were recently shown to have increased dependency on SR-BI for entry, suggesting that the virus modulates NAb sensitivity at the cost of a more complicated entry pathway [8, 23, 24]. As HVR1 has been shown to modulate binding of E2 to SR-BI [20], unraveling interactions between HVR1 and other parts of the envelope proteins might be key to understand the link between immune evasion and viral entry.

Here, we found that cell culture recombinants (HCVcc) with E1/E2 from H77 (genotype 1a) in which HVR1 was swapped from strains DH5 (genotype 1b), SA13 (genotype 5a) or HK6a (genotype 6a), as well as HCVcc J4 (genotype 1b) with HVR1 from S52 (genotype 3a), were unable to produce infectious particles in Huh7.5 cells. Studying the culture adaptation of these HVR1-swapped recombinants, we identified isolate-specific envelope protein substitutions that specifically ameliorated loss of viral infectivity. These substitutions decreased viral entry dependency on SR-BI and generally increased NAb sensitivity. However, this NAb sensitizing effect only partly correlated with NAb binding to extracted E1/E2 protein and immunoprecipitated HCV particles for J4 recombinants and not for H77 recombinants. Our study highlights important shortcomings of current envelope protein model systems to study antibody binding while contributing to the understanding of HVR1-mediated protection of cross-genotype conserved NAb epitopes and shedding light on interactions between HVR1 and the remaining part of E1/E2.

## Results

### Culture-adapted HVR1-swapped H77 HCVcc recombinants acquire E1 and E2 substitutions

**Fig 1:**
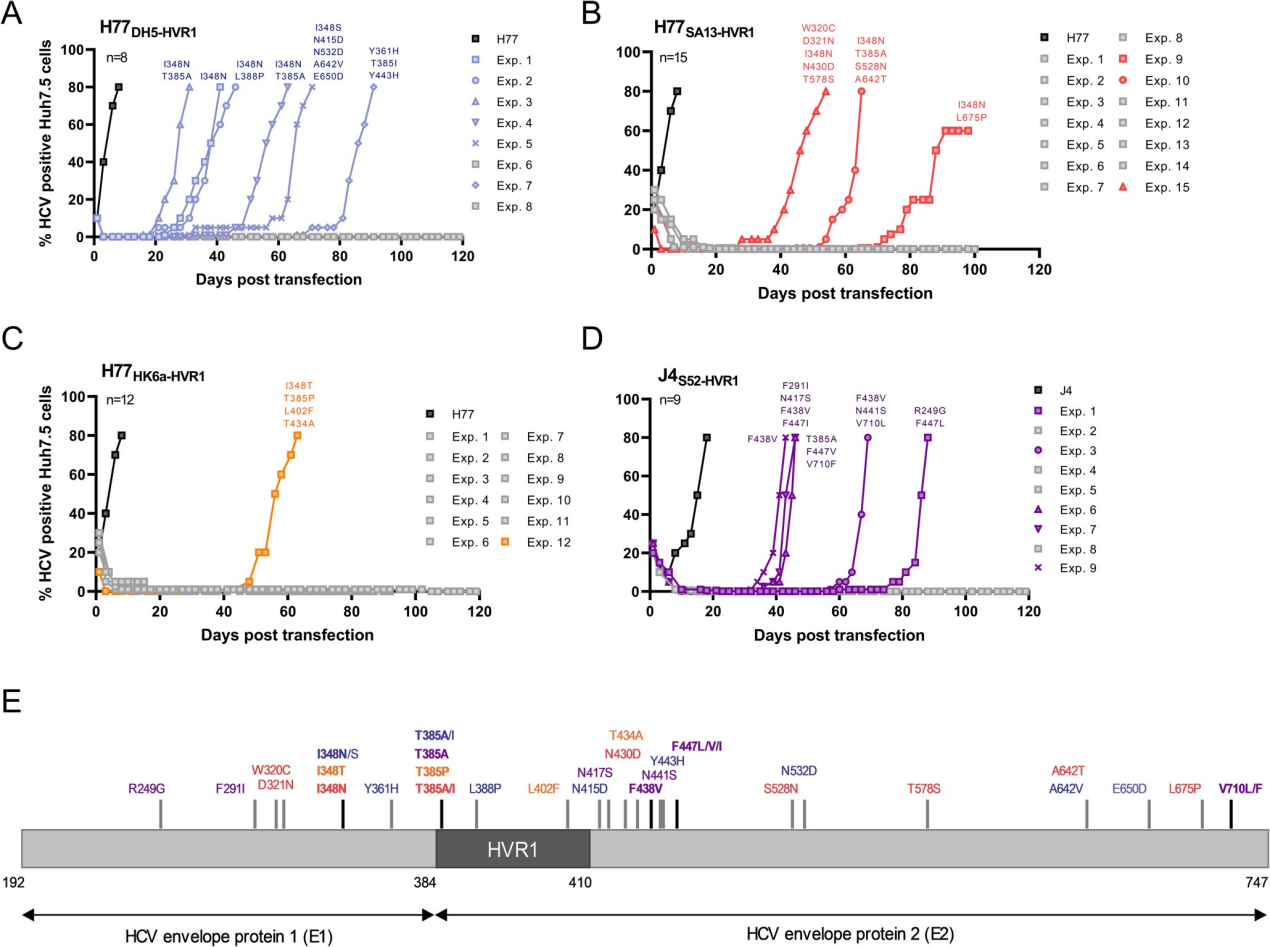
Insertion of HVR1 from DH5, SA13 and HK6a into H77 or S52 HVR1 into J4 attenuates the recombinant HCVcc. (A-D) Adaptation of the HVR1-swapped viruses in Huh7.5 cells during the indicated time period. The cells were transfected with the indicated *in vitro* transcribed HCV RNA of the indicated recombinants. Viral spread was monitored by immunostainings of infected cells and sequence analysis was performed on amplified envelope protein encoding nucleotide sequences of the cell culture adapted HVR1-swapped recombinants. Spread of a representative parental H77 (A-C) and J4 (D) recombinant is shown in black in all panels. Only coding mutations are depicted. Gray squares indicate cell cultures that were closed due to infection suppression, as evidenced by the absence of antigen-positive cells for at least two weeks. (E) Schematic overview of the envelope proteins with the identified coding mutations color-coded according to which HVR1-swapped recombinant they were identified in (colours correspond to those shown in panels A-D). Amino acid positions are based on H77 abs. ref. (Genbank #AF009606); HVR1 corresponds to positions 384–410.

To study the role of HVR1 in the HCV life cycle, we previously generated JFH1-based HCV recombinants with Core-NS2 of H77 (genotype 1a) with HVR1-swapped from isolates of genotype 1–6 [8]. HCV RNA transfection of Huh7.5 cells with the HVR1-swapped recombinants revealed that H77 with HVR1 from DH5 (genotype 1b), SA13 (genotype 5a) and HK6a (genotype 6a) (named H77_DH5-HVR1_, H77_SA13-HVR1_, and H77_HK6a-HVR1_, respectively), were highly attenuated, displaying inefficient or non-existent spread in Huh7.5 cells (Fig 1). However, monitoring long-term cultures of Huh7.5 cells transfected with these recombinants, we observed a late increase in HCV positive cells after an eclipse phase of 30–90 days in six out of eight H77_DH5-HVR1_ transfections (Fig 1A), three out of 15 H77_SA13-HVR1_ transfections (Fig 1B) and one out of 12 H77_HK6a-HVR1_ transfections (Fig 1C). We extracted HCV RNA from supernatants collected at the peak of infection (>80% HCV positive cells) and sequenced the envelope protein-coding sequence. Sequence analysis consistently confirmed the HVR1 swap and identified putative compensatory envelope protein substitutions (Fig 1A–1C and S1 Table); coding envelope changes are summarized in Fig 1E. Interestingly, irrespective of HVR1 sequence, the recovered HVR1-swapped H77 recombinants acquired substitutions at positions 348 (I348N/S/T; E1) and 385 (T385A/P/I; HVR1 of E2), either alone or in combination. This suggested an HVR1-sequence independent compensatory adaptive pathway to overcome decreased infectivity for attenuated HVR1-swapped H77 recombinants.

### Envelope substitutions at position 348 (E1) and 385 (E2) fully restore the infectivity of attenuated HVR1-swapped H77 HCVcc recombinants

**Fig 2:**
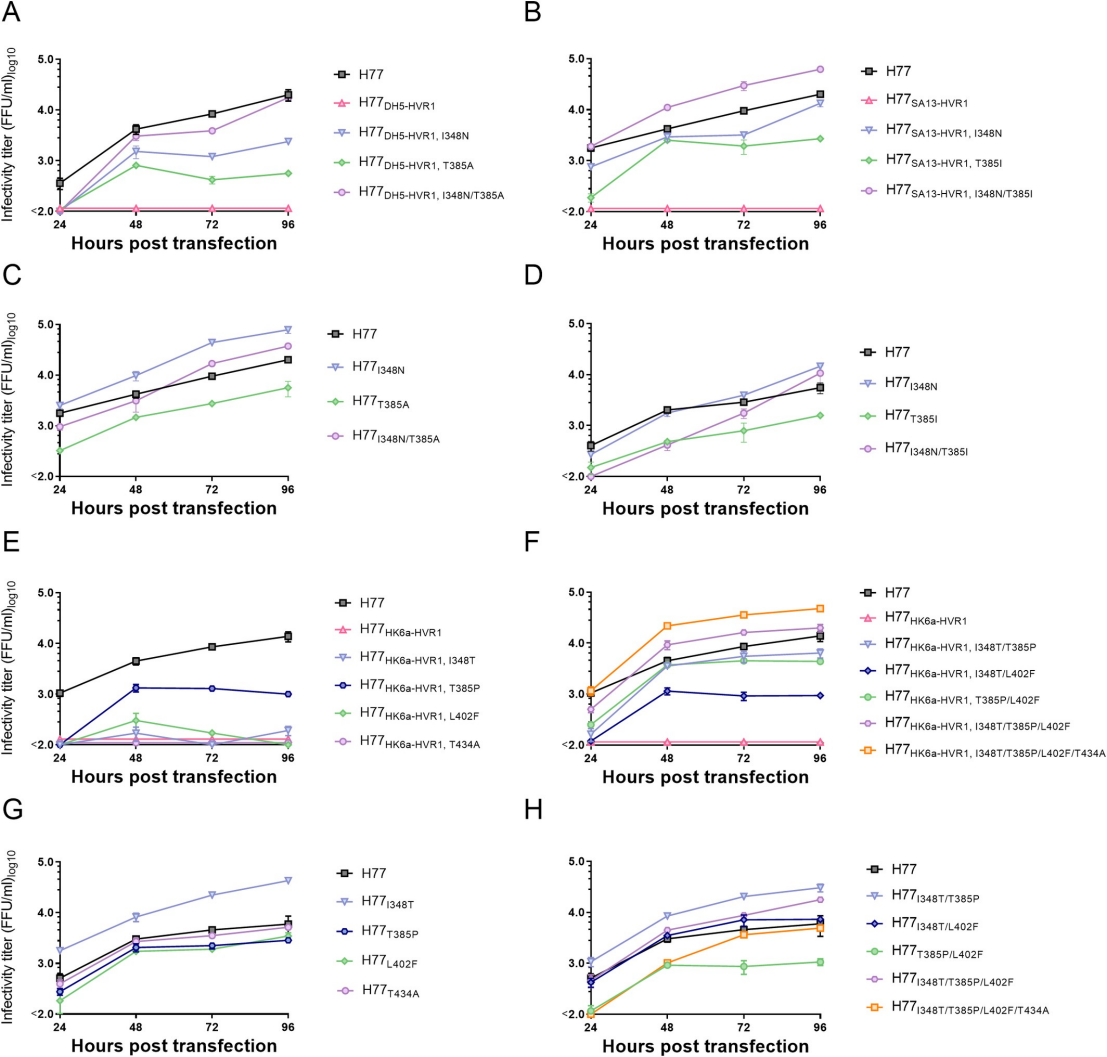
HVR1-swapped H77 recombinants require specific substitutions in E1 and HVR1 of E2 to restore infectivity. Huh7.5 cells were transfected with *in vitro* transcribed HCV RNA of the indicated recombinants. Supernatants were collected 24, 48, 72 and 96 hours post transfection, and HCV infectivity titers were determined. At each timepoint, infectivity titers are represented as a mean of three technical replicates. Error bars represent standard deviation. Lower level of quantification was 100 FFU/ml. The substitutions are numbered according to H77 abs. ref. (GenBank #AF009606). The data shown is a representative experiment out of at least 2.

For H77_DH5-HVR1_ and H77_SA13-HVR1_, we selected the most frequently observed envelope substitutions for further analysis. These were I348N and T385A for H77_DH5-HVR1_ and I348N and T385I for H77_SA13-HVR1_ (S1 Table). The substitutions singly or combined were introduced into the original H77 and the relevant HVR1-swapped H77 recombinants. We tested their effect on viral fitness by HCV RNA transfection of Huh7.5 cells, followed by monitoring the spread and release of infectious particles every 24 hours. Viral spread was monitored by immunostaining to detect the number of HCV positive cells, and the release of viral particles was measured by focus forming units (FFU) titration of cell culture supernatant collected 24, 48, 72 or 96 hours post transfection. The E1 substitution I348N increased infectivity above the assay cut-off by increasing titers of both H77_DH5-HVR1_ and H77_SA13-HVR1_ by at least 14- to 21-fold (Fig 2A and 2B), while modestly increasing the infectivity of H77 (2.6–4.0-fold higher titers) (Fig 2C and 2D). Conversely, while T385A increased the infectivity of H77_DH5-HVR1_ 7-fold and T385I increased the infectivity of H77_SA13-HVR1_ 14-fold, both substitutions decreased the infectivity of H77 by ~5-fold (Fig 2A–2D). The combination of substitutions I348N and T385A fully restored the infectivity of H77_DH5-HVR1_ and the combination of I348N and T385I fully restored the infectivity of H77_SA13-HVR1_, with titers comparable to H77 (Fig 2A and 2B). In addition, I348N ameliorated the attenuating effect of T385A/I in H77 (Fig 2C and 2D), indicating that the negative effect of T385A/I was either corrected for by I348N or rendered less impactful. Thus, we demonstrated that the adaptive effect of T385A/I depended on I348N and that the combination of these substitutions restored the infectivity and efficient virus spread of H77_DH5-HVR1_ and H77_SA13-HVR1_.

Only one out of 12 H77_HK6a-HVR1_ transfections spread in culture (Fig 1C). HCV RNA sequencing revealed four putative adaptive envelope substitutions, I348T, T385P, L402F and T434A, that we introduced alone or in combinations into H77_HK6a-HVR1_ and H77. T385P increased infectivity above the lower assay cut-off by increasing titers of H77_HK6a-HVR1_ at least 10-fold, while the three other substitutions alone didn’t result in measurable infectivity titers of H77_HK6a-HVR1_ (Fig 2E). However, when combinations of these substitutions were introduced into H77_HK6a-HVR1_, they did increase infectivity (Fig 2F). I348T, T385P and T434A combined and all four envelope substitutions combined increased infectivity of H77_HK6a-HVR1_ to levels similar to that of H77. Only I348T increased the infectivity of H77 (8-fold), whereas the other substitutions had negligible effects (Fig 2G). Interestingly, T385P combined with I348T increased H77 titers 7-fold, while the combination of T385P and L402F decreased H77 infectivity titers 8-fold (Fig 2H). Finally, all four substitutions introduced together into H77 had no significant effect on infectivity. Thus, we concluded that while the substitutions I348T and T385P were compensatory for H77_HK6a-HVR1_, they were insufficient to fully restore infectivity by themselves_._

To assess whether any of the observed envelope substitutions were general cell culture adaptive mutations for H77 HCVcc recombinants, we transfected three independent Huh7.5 cell cultures with parental H77 Core-NS2 recombinants. Supernatants taken 72 hours post transfection were then passaged on naïve Huh7.5 cells twice until they infected at least 80% of cells (1^st^ passage spread in 12 days and 2^nd^ passage spread in 9 days). HCV RNA was extracted from 1^st^ and 2^nd^ passage at peak of infection and the envelope protein sequences were determined. None of the cultures acquired substitutions in 1^st^ passage. In 2^nd^ passage, one of three culture passages of H77 had not acquired any envelope protein substitutions, whereas we observed changes for the two remaining cultured viruses; at position Y361H (E1) or at position S478G (E2). As these did not overlap with the changes described above for HVR1-swapped H77 recombinants, this indicated that the HVR1-swap adaptive envelope substitutions were specifically compensating the attenuating effect of the chimeric HVR1 sequence.

**Fig 3:**
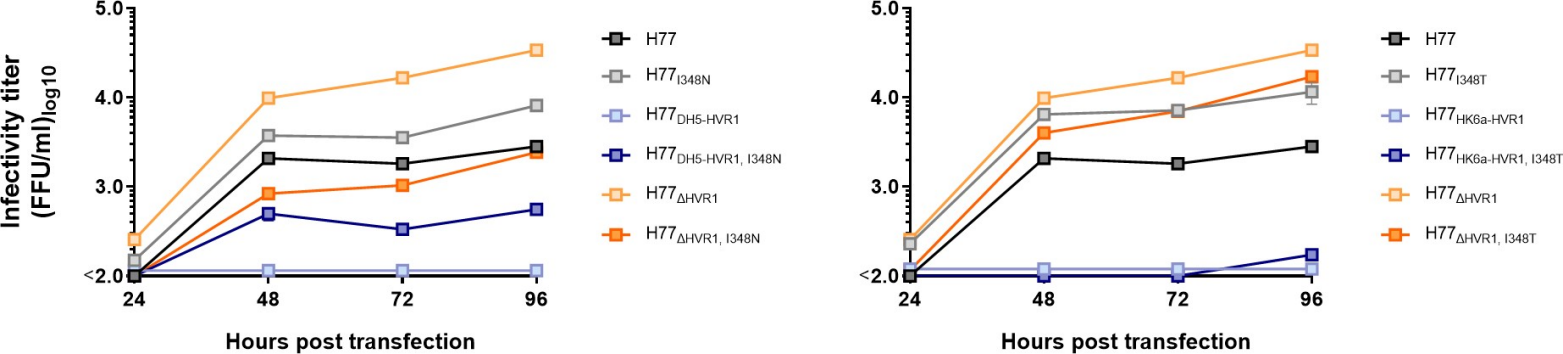
The substitutions I348N and I348T specifically compensate attenuated H77 HVR1-swapped recombinants. Huh7.5 cells were transfected with *in vitro* transcribed HCV RNA of the indicated recombinants. Supernatants were collected 24, 48, 72 and 96 hours post transfection, and HCV infectivity titers were determined. At each timepoint, infectivity titers are represented as a mean of three technical replicates. Error bars represent standard deviation. Lower level of quantification was 100 FFU/ml. The substitutions are numbered according to H77 abs. ref. (GenBank #AF009606).

In order to study if the E1 substitutions at position 348 that increased infectivity of the attenuated HVR1-swapped H77 recombinants were important for functional HVR1-E1/E2 interactions, we tested the effect of substitutions I348N and I348T on infectivity of H77_ΔHVR1_. As described above, we tested viral fitness by HCV RNA transfection of Huh7.5 cells, followed by monitoring the spread and release of infectious particles every 24 hours for 96 hours. Interestingly, we found that I348N and I348T decreased infectivity of H77_ΔHVR1_ by 11-fold or 3-fold, respectively (Fig 3), supporting that the adaptive effect of these substitutions was specifically linked to HVR1 functionality.

### The infectivity of attenuated HVR1-swapped H77 HCVcc recombinants is restored by reversion of N-terminal HVR1 residues

**Fig 4:**
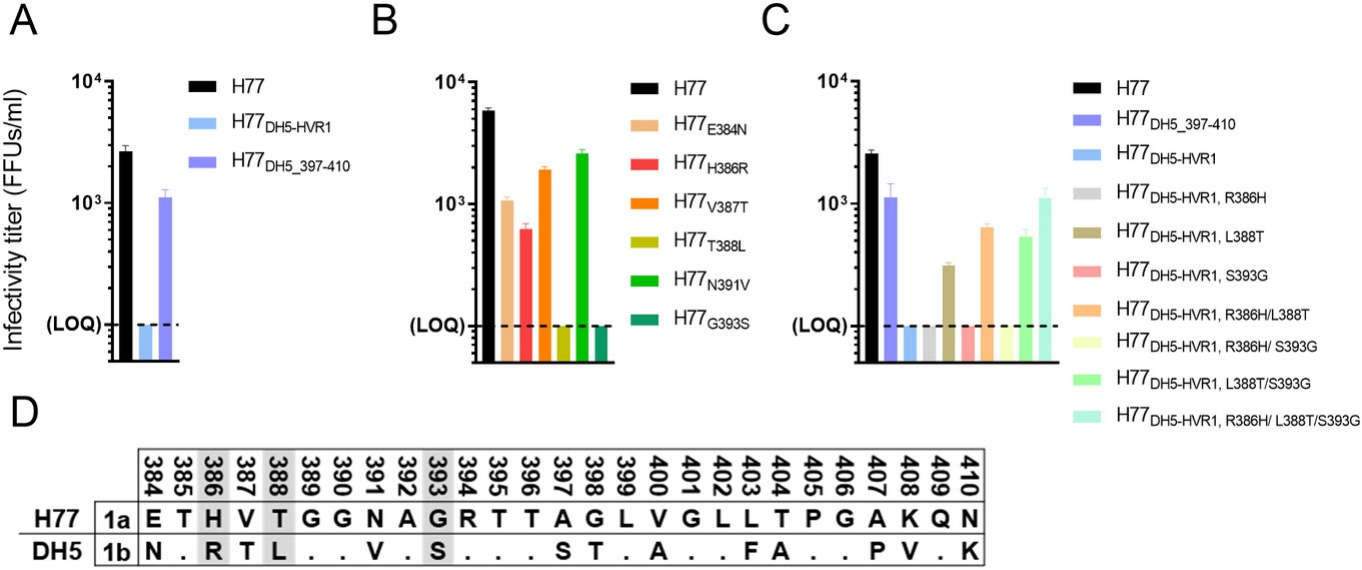
Infectivity of H77_DH5-HVR1_ is restored by introducing the H77 residue at the N-terminal HVR1 positions 386, 388 and 393. (A-C) Huh7.5 cells were transfected with in vitro transcribed HCV RNA of the indicated recombinants. Supernatants were collected 24, 48, 72 and 96 hours post transfection, and HCV infectivity titers were determined. The infectivity titers of the recombinants are shown at 72 hours post transfection as a mean of three technical replicates (complete data sets are shown in S1 Fig). Error bars represent standard deviation. Lower level of quantification was 100 FFU/ml. The substitutions are numbered according to H77 abs. ref. (GenBank #AF009606). (D) Alignment of HVR1 from HCV genotype 1b isolate DH5 against genotype 1a H77 reference genome (GenBank accession no. AF009606; Mega 7). Dots indicate homology with H77. The data shown is a representative experiment out of at least 2. Positions in HVR1 shown to be responsible for viral attenuation in H77 HVR1 swap recombinants are highlighted in grey.

Next, we investigated which part of the heterologous HVR1 sequence was responsible for the attenuating effect in H77 HCVcc. Focusing on DH5-HVR1, we tested whether the observed attenuating effect was caused by the C-terminal part of this HVR1 sequence by introducing positions 397 to 410 from DH5 into H77. H77_DH5_397–410_ was viable with titers only 2.4-fold lower than H77 (Figs 4A and S1A), indicating that residues in the N-terminal part of DH5-HVR1 were the cause of H77_DH5-HVR1_ attenuation. As DH5 differed from H77 at six amino acid positions in this part of HVR1 (Fig 4D), we substituted each of the six residues singly into H77. We found that L388 or S393 completely attenuated viral spread, while the introduction of the other DH5 residues resulted in minor decreases in infectivity, with R386 having the biggest effect (Figs 4B and S1B). To test if R386, L388 or S393 were primarily responsible for the attenuating effect of DH5-HVR1 in H77, these residues were reverted in H77_DH5-HVR1_ either singly or in combination. Interestingly, we observed that the substitution L388T increased the infectivity of H77_DH5-HVR1_ and that the combination of the substitutions R386H, L388T and S393G in H77_DH5-HVR1_ reproduced the fitness of H77_DH5_397–410_ (Figs 4C and S1C). Thus, our data demonstrated that positions in the N-terminal part of HVR1 determined the compatibility of HVR1 with the remaining part of H77 E1/E2.

### The envelope substitutions I348N and T385A increase neutralization susceptibility of H77 HCVcc recombinants to broad NAbs AR3A and AR4A

To study the identified HVR1-swap adaptive envelope protein substitutions in greater detail, we generated envelope protein sequence-confirmed 1^st^ passage virus stocks of the HVR1-swapped recombinants and the original viruses with selected compensatory substitutions (S2 Table). For some of the less adapted recombinants, we were not able to produce virus stocks without additional substitutions. This applied to H77_T385A_ that acquired either A343V or Y718C and H77_T385I_ that acquired I348S or Y718C. We were able to produce a clean virus stock of H77_T385P_, but only at the second attempt, as the first culture also acquired I348S. H77_DH5-HVR1, I348N_ and H77_SA13-HVR1, I348N_, acquired T385P or T385A, respectively, and H77_SA13-HVR1, T385I_, acquired G350V, thus, confirming the importance of the combination of substitutions at E1 around positions 348 and a substitution in HVR1 at 385. These virus stocks were excluded from further analysis.

**Fig 5:**
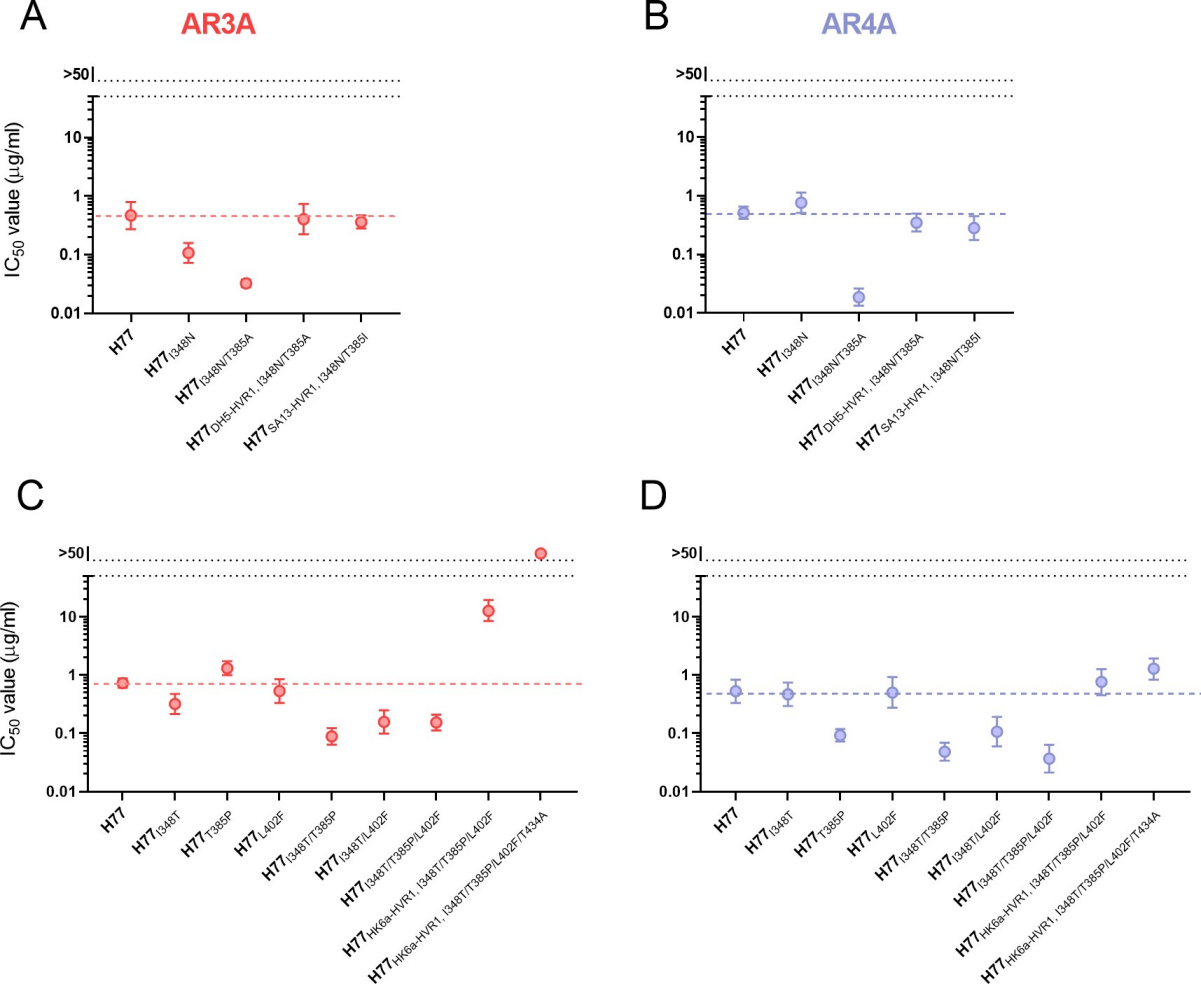
**E1/E2 substitutions identified in HVR1-swapped H77 recombinants increase sensitivity of H77 to AR3A and AR4A.** Neutralization by the bNAbs AR3A (A and C) and AR4A (B and D) of the indicated HCV recombinants. The viruses were incubated in four technical replicates with a 5-fold dilution series of the bNAbs, starting at 50 μg/ml, along with eight technical replicates of virus only. 48 hours post-infection the cells were immunostained for HCV antigen, and the number of FFUs/well was counted and normalized to the mean count of wells with virus only. Each dot represents the mean IC_50_ value of the indicated recombinants. Error bars represent the 95% confidence intervals (CI_95_). The colored broken line represents the IC_50_ value of unmodified H77. The data was analyzed using three-parameter dose-response with the top value set to 100 and lower value set to 0, to calculate IC_50_ and CI_95_ using GraphPad Prism v8.0.0.

Using the virus stocks without undesirable substitutions, we performed dose-response FFU reduction neutralization assays with the broadly neutralizing monoclonal antibodies (bNAbs) AR3A [25] and AR4A [26]. All viruses exhibited dose-dependent neutralization. H77, H77_DH5-HVR1, I348N/T385A_ and H77_SA13-HVR1, I348N/T385I_ were similarly sensitive to AR3A and AR4A (Fig 5A and 5B). In contrast, H77_I348N_ was significantly more sensitive to AR3A than H77, and this was even more pronounced for H77_I348N/T385A_ (Fig 5A). Interestingly, I348N did not affect H77 sensitivity to AR4A, while the combination of I348N and T385A significantly increased the sensitivity of H77 to AR4A (Fig 5B).

The H77_HK6a-HVR1, I348T/T385P/L402F_ and H77_HK6a-HVR1, I348T/T385P/L402F/T434A_ recombinant viruses were both significantly more resistant to AR3A compared with H77 (Fig 5C). However, these recombinants were all similarly sensitive to AR4A (Fig 5D). For H77, the substitutions I348T and L402F had little or no effect on the sensitivity of H77 to AR3A and AR4A. In contrast, T385P increased sensitivity of H77 to AR4A but did not affect AR3A sensitivity. I348T combined with either T385P or L402F or all three substitutions combined increased sensitivity to AR3A and AR4A (Fig 5C and 5D). Overall, the data show that the compensatory substitutions, identified for H77_DH5-HVR1_, H77_SA13-HVR1_ and H77_HK6a-HVR1_, all increased neutralization sensitivity of H77 to both AR3A and AR4A, while the H77 HVR1-swapped recombinants were either as sensitive or less sensitive than H77, perhaps suggesting that the swap of HVR1 could be rendering the virus less sensitive to NAbs.

### Culture adapted HVR1-swapped J4 HCVcc recombinants acquire E2 substitutions

To study the effect of swapping divergent HVR1s into other isolates than H77, we generated JFH1-based HCV recombinants of J4 Core-NS2 (genotype 1b) with HVR1 from H77 (genotype 1a), J6 (genotype 2a) and S52 (genotype 3a). To test viral infectivity of the J4 HVR1-swapped recombinants, we performed HCV RNA transfection of Huh7.5 cells. We found that J4_H77-HVR1_ and J4_J6-HVR1_ were viable with titers 5-fold lower than J4, whereas J4_S52-HVR1_ was highly attenuated, displaying inefficient or non-existent spread in Huh7.5 cells (S2 Fig). Monitoring long-term cultures of Huh7.5 cells transfected with J4_S52-HVR1_, we observed that in five out of nine transfections, viral spread occurred following an eclipse phase of 46 to 88 days (Fig 1D). Supernatant was collected at the peak of infection (>80% HCV positive cells), and the HCV envelope protein-coding sequences were determined (coding envelope mutations are summarized in Fig 1E and S3 Table). Only one out of the five recovered viruses acquired a substitution in HVR1 (T385A, also observed in HVR1-swapped H77, S3 Table exp. 3). Interestingly, substitutions observed in adapted J4_S52-HVR1_ cultures were found at positions that differed from those observed in adapted HVR1-swapped H77 cultures, suggesting a different pathway of adaptation for attenuated HVR1-swapped J4 recombinants.

We consistently observed either the substitution F438V (E2) or one of three changes at position 447: F447L, F447V, or F447I (E2). In fact, all adapted J4_S52-HVR1_ viruses had substitutions at one of these positions, but never both, suggesting that one of the substitutions is enough to regain infectivity and perhaps that their beneficial effects on virus infectivity were mutually exclusive. Substitutions at these two positions were consistently observed in combination with other envelope protein substitutions, suggesting that additional substitutions could be required for full recovery of viral infectivity. For one adapted J4_S52-HVR1_ culture, we observed only partial nucleotide changes (Fig 1D and S3 Table exp. 5). This suggested multiple adapted viral lineages, which we confirmed was the case by performing clonal analysis of the E1E2 coding sequence from TOPO XL cloned PCR products of the exp. 5 culture supernatant. This permitted us to group the observed substitutions into separate lineages (S4 Table), showing that the lineages had acquired either F438V or F447I.

### HCV envelope protein substitutions fully restore the infectivity of attenuated HVR1-swapped J4 HCVcc recombinants

**Fig 6:**
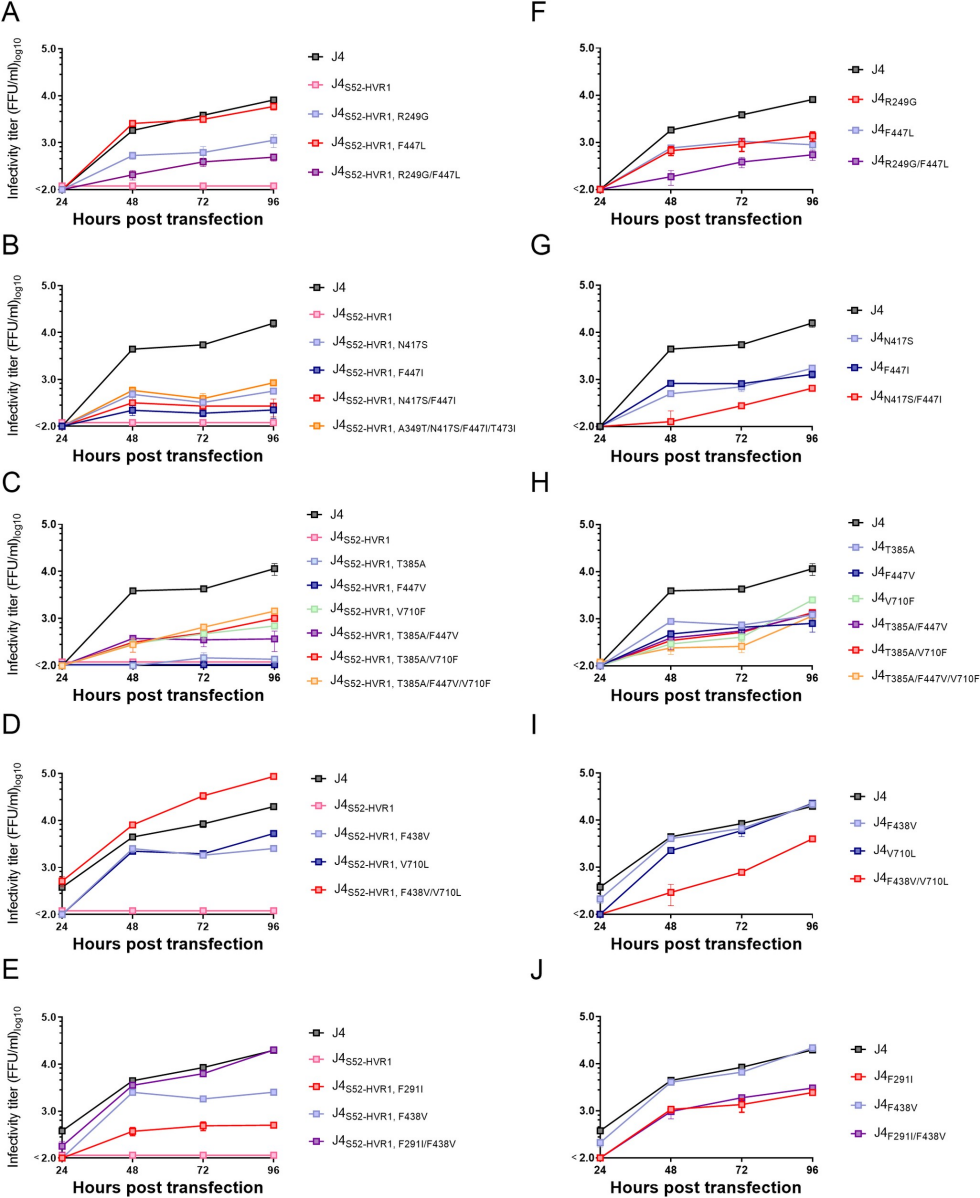
J4_S52-HVR1_ requires specific substitutions in E2 to regain infectivity. Huh7.5 cells were transfected with *in vitro* transcribed HCV RNA of the indicated recombinants. Supernatants were collected 24, 48, 72 and 96 hours post transfection and HCV infectivity titers were determined. At each timepoint, infectivity titers are represented by the mean of three technical replicates. Error bars represent standard deviation. Lower level of quantification was 100 FFU/ml. The substitutions are numbered according to H77 abs. ref. (GenBank #AF009606). The data shown is a representative experiment out of at least 2.

Based on the sequence data of the recovered J4_S52-HVR1_ recombinants, adaptive substitutions were commonly found at two E2 positions outside HVR1: F438V or F447L/V/I. These substitutions were introduced singly and in combinations with other observed envelope substitutions into the original J4 and HVR1-swapped J4_S52-HVR1_ recombinants. These were F438V in combination with F291I (E1) or V710L (E2), F447L with R249G (E1), F447I with N417S (E2), and finally F447V with T385A (HVR1, E2) or V710F (E2). As described for H77, we tested the effect of the substitutions on viral fitness by HCV RNA transfection of Huh7.5 cells. We found that F447L alone fully restored the infectivity of J4_S52-HVR1_ (Fig 6A). Interestingly different substitutions at the same position (F447I and F447V) had little or no effect on the infectivity of J4_S52-HVR1_ (Fig 6B and 6C). When F447I was introduced together with N417S (Fig 6B) or when F447V was introduced together with T385A or V710F (Fig 6C), the substitutions were still not able to restore infectivity despite repeat transfections. In J4, F447L, F447I or F447V all decreased the infectivity ~9-fold (Fig 6F–6H), suggesting that the effect of F447L was HVR1-dependent. F438V was adaptive in J4_S52-HVR1_, but the additional substitutions F291I or V710L were required to fully restore the infectivity of the recombinant (Fig 6D and 6E). Interestingly, the same substitutions had different effects in J4, for which F438V and V710L did not affect infectivity (Fig 6I and 6J) and F291I decreased infectivity of J4 7-fold (Fig 6J). In J4, the combination of F438V with either F291I or V710L decreased the infectivity 7- to 8-fold (Fig 6I and 6J), again indicating HVR1-dependent effects.

As for the H77 HVR1-swapped recombinants, we assessed whether the envelope substitutions observed for J4_S52-HVR1_ would also appear in adaptation of parental J4 Core-NS2 recombinant HCVcc. Thus, we transfected three independent Huh7.5 cell cultures with J4 HCV RNA. We collected supernatant 72 hours post transfection and used this to passage J4 onto naive Huh7.5 cells twice until they infected at least 80% of the cells (first passage spread in 20 days and 2^nd^ passage spread in 18 days). At the peak of infection of 1^st^ and 2^nd^ passage, we extracted HCV RNA and determined the envelope protein sequences. After 1^st^ passage, none of the cultures acquired substitutions within the envelope, but after 2^nd^ passage all three cultured J4 acquired a substitution at position N576D. Substitutions at this position have also been described by others for cell culture adaptation of J4/JFH1 [27]. The substitution does not overlap with the substitutions found in the J4 HVR1-swapped recombinants, which indicated that the HVR1-swap adaptive envelope substitutions were specifically compensating the attenuating effect of HVR1 swap into J4.

**Fig 7:**
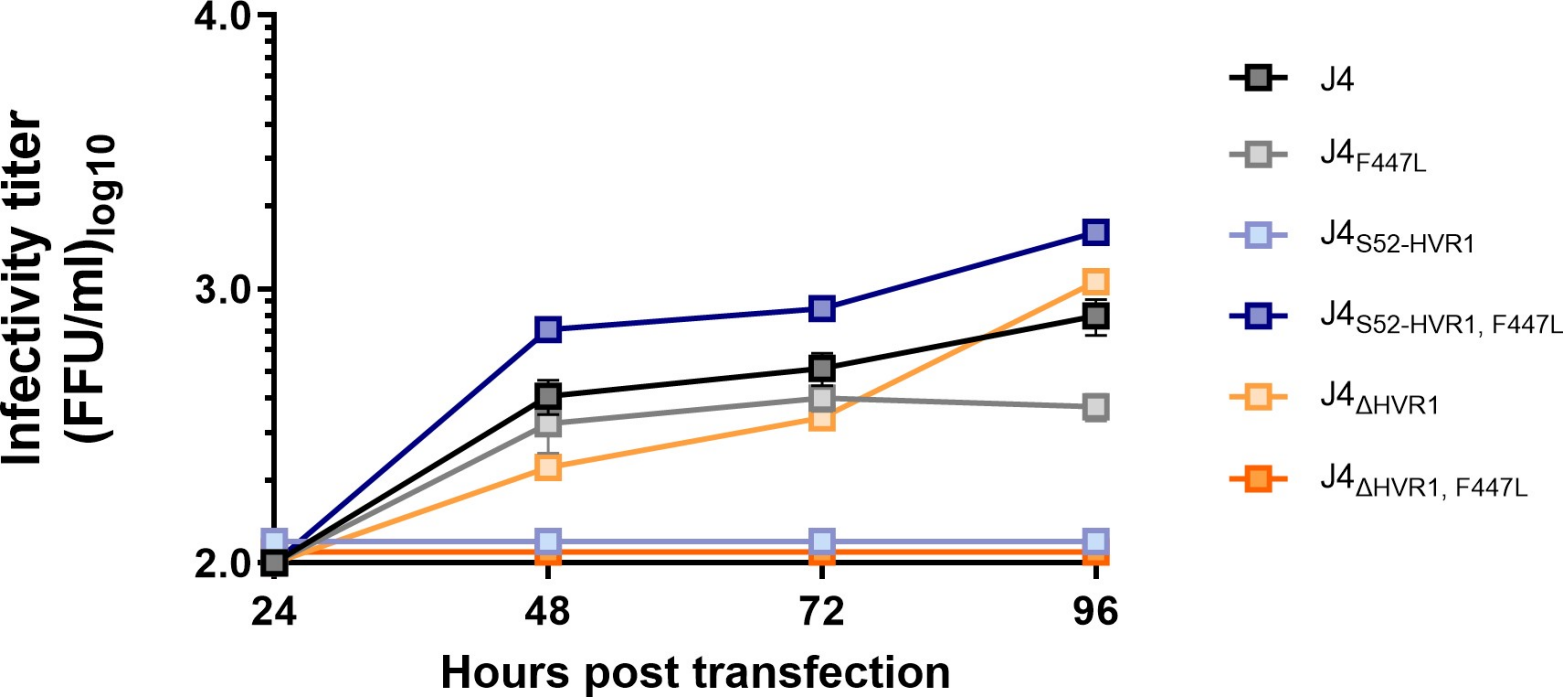
The substitution F447L is adaptive for J4_S52-HVR1_, while it decreases infectivity of parental J4 and J4_ΔHVR1_ HCVcc. Huh7.5 cells were transfected with *in vitro* transcribed HCV RNA of the indicated recombinants. Supernatants were collected 24, 48, 72 and 96 hours post transfection and HCV infectivity titers were determined. At each timepoint, infectivity titers are represented by the mean of three technical replicates. Error bars represent standard deviation. Lower level of quantification was 100 FFU/ml. The substitutions are numbered according to H77 abs. ref. (GenBank #AF009606).

To study if the substitution, F447L, that was found to fully restore infectivity of J4_S52-HVR1_, specifically compensated non-functional HVR1-E1/E2 interactions in J4, we tested the effect of F447L on infectivity of J4_ΔHVR1_. Interestingly, we found that F447L attenuated J4_ΔHVR1_ with infectivity titers below the level of detection (Fig 7), supporting that the adaptive effect of this substitution was specifically linked to HVR1 functionality for this isolate.

As some of the putative compensatory envelope substitutions identified in adapted J4_S52-HVR1_ cultures were not, neither alone nor in combination, able to restore the infectivity of the attenuated HVR1-swapped J4_S52-HVR1_ recombinant (Fig 6B and 6C), we sequenced the full ORF of these adapted recombinants. This revealed several additional putative compensatory substitutions (S5 Table). These substitutions clustered within the HCV proteins, NS3 and NS5A, which is similar to what was previously observed in general adaptation studies of JFH1-based J4 Core-NS2 recombinants [27]. Interestingly, this potential contribution from cell culture adaptive mutations in non-E1E2 proteins only seemed salient for J4_S52-HVR1_, as the infectivity of H77_DH5-HVR1_, H77_SA13-HVR1_ and H77_HK6a-HVR1_ was fully restored upon introduction of E1E2 substitutions, perhaps indicating that the original J4 recombinant is less adapted to cell culture.

### The envelope substitutions found in J4_S52-HVR1_ HCVcc significantly increase the sensitivity of J4 to AR3A while having negligible or opposite effects on AR4A sensitivity

**Fig 8:**
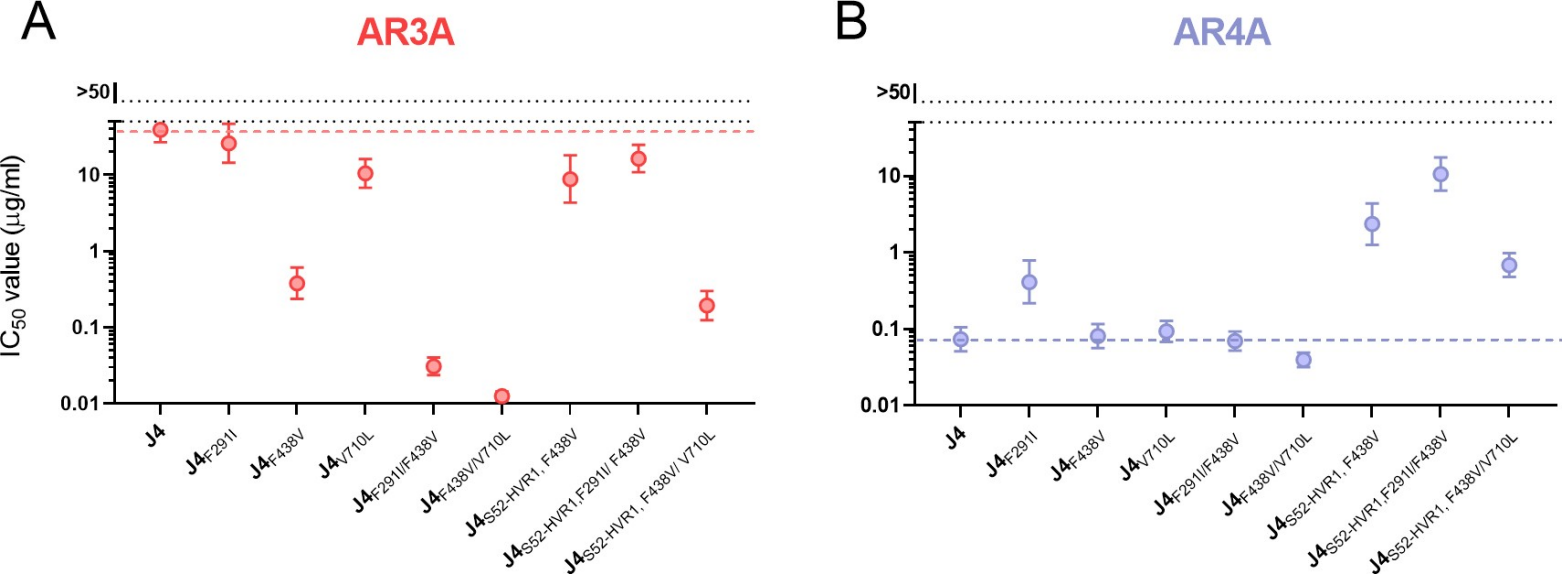
Envelope protein substitutions increase sensitivity of J4 HCVcc to AR3A with little effect on AR4A sensitivity. Neutralization by the bNAbs (A) AR3A and (B) AR4A of the indicated recombinants. The viruses were incubated in four technical replicates with a 5-fold dilution series of the bNAbs, starting at 50 μg/ml, along with eight technical replicates of virus only. 48 hours post-infection the cells were immunostained for HCV antigen, and the number of FFUs/well was counted and normalized to the mean count of wells with virus only. Each dot represents the mean IC_50_ value of the indicated recombinants. Error bars represent the 95% confidence intervals (CI_95_). The colored broken line represents the IC_50_ value of unmodified J4. The data was analyzed using three-parameter dose-response with the top value set to 100 and lower value set to 0, to calculate IC_50_ and CI_95_ using GraphPad Prism v8.0.0.

To investigate the effect of the substitutions identified in the J4_S52-HVR1_ adapted viruses, we generated envelope protein sequence-confirmed 1^st^ passage virus stocks of J4_S52-HVR1_ and J4 with selected compensatory substitutions (S2 Table). One low-titer recombinant, J4_S52-HVR1, V710L_, acquired the additional envelope substitution T434P (E2) (also observed in the adapted H77_HK6a-HVR1_ recombinant), and was thus excluded from further analysis. Using the generated virus stocks, we performed dose-response FFU reduction neutralization assays with AR3A and AR4A. For AR3A, we observed that J4_S52-HVR1, F438V_ was more sensitive than J4 and that the introduction of V710L further increased the sensitivity of this recombinant (J4_S52-HVR1, F438V/V710L_ was 200-fold more sensitive than J4) (Fig 8A). F438V also drastically increased the sensitivity of J4 to AR3A, whereas V710L only had a minor sensitizing effect and F291I had no effect, thus supporting that the substitution at position 438 was the main contributor to the increase in AR3A sensitivity. However, the combination of F438V with either F291I or V710L increased the sensitivity of J4 against AR3A even further (more than 1000-fold), suggesting that the contribution of these substitutions to AR3A sensitivity was dependent on F438V. Conversely, for AR4A, which targets an epitope with contact residues in both E1 and E2, J4_S52-HVR1_ with the different combinations of substitutions were all more resistant compared to J4 (Fig 8B). However, for J4, only F291I increased AR4A resistance, while the other substitutions, singly or in combination, had minor or negligible effects. Additionally, we tested the effect of the substitutions on the sensitivity to another monoclonal bNAb, AR5A, targeting an epitope with contact residues in both E1 and E2 different from AR4A [26]. The effect of substitutions on J4 and J4_S52-HVR1_ sensitivity to AR5A (S3 Fig) were similar to the effects we observed for AR4A, indicating that bNAbs targeting epitopes in the interface between E1 and E2 were less affected by these substitutions.

### Detergent extracted HCV E1/E2 protein complexes do not consistently recapitulate the neutralization of virion-associated E1/E2

**Fig 9:**
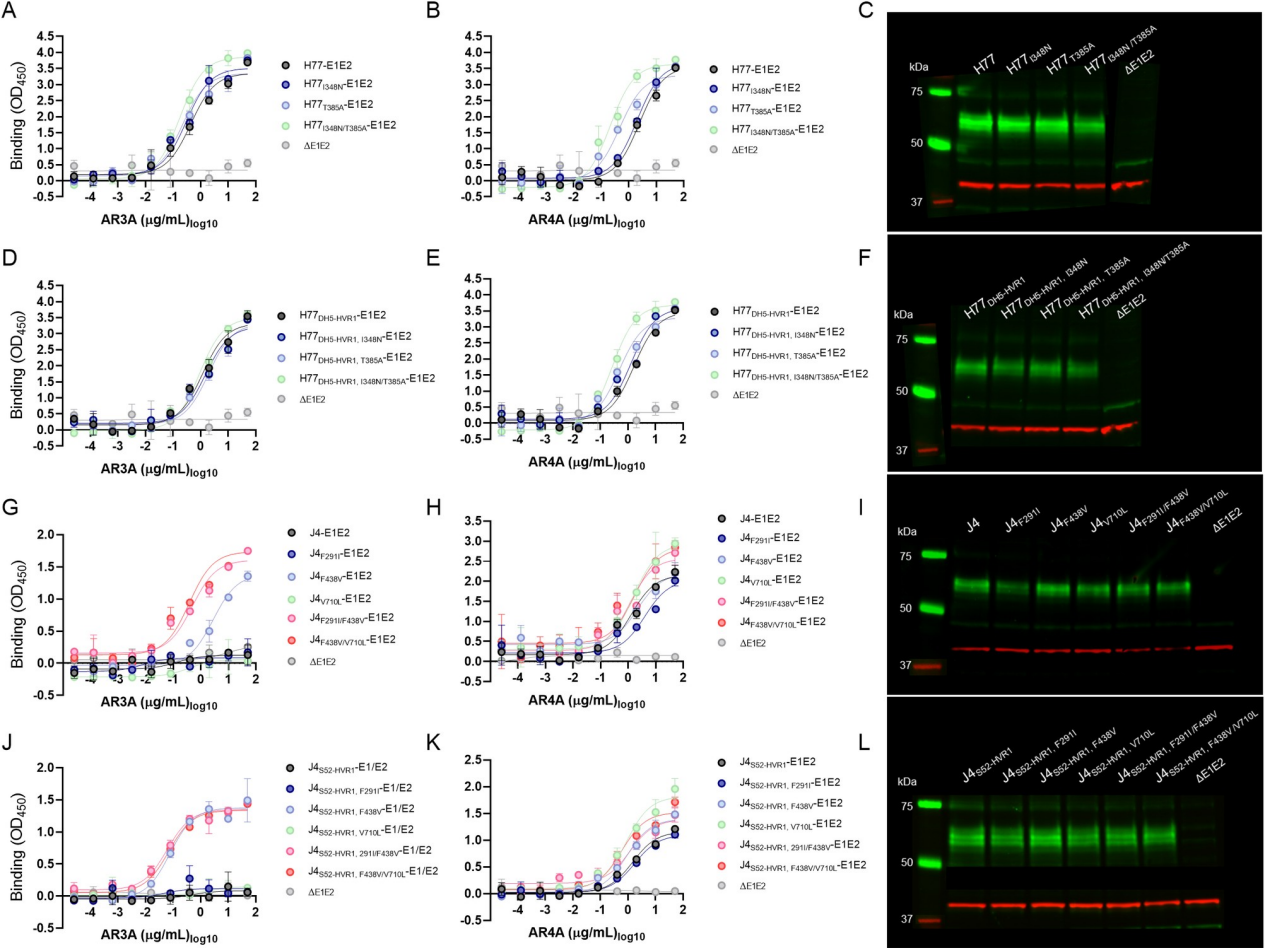
HVR1 swaps and associated adaptive envelope mutations have divergent effects on antibody binding to extracted E1/E2 protein. (A-B, D-E, G-H and J-K) Binding curves for dilution series of monoclonal antibodies (mAbs) AR3A and AR4A in ELISA to the indicated extracted E1/E2 proteins. Cell lysates containing E1/E2 protein were added to lectin-coated ELISA plates and incubated overnight. mAbs were added in 5-fold dilution series for a 2-hour incubation. The binding was detected with an HRP-conjugated anti-human IgG secondary antibody. TMB Stabilized Chromagen substrate (Thermo Fisher Scientific) was added, and absorbance was measured at 450 nm. Each data point represents a mean of two technical replicates with error bars representing standard derivation. The data was analysed using three-parameter nonlinear regression using Graphpad Prism v8.0.0. The data shown is a representative experiment out of at least 2. (C, F, I and L) Quantification of E2 protein in collected cell lysates. Equivalent volumes of the cell lysates used in panels A-B, D-E, G-H and J-K were run on SDS-PAGE, and E2 protein was visualized in a fluorescent western blot using the mAb, AP33. β-actin was visualized as a loading control. ΔE1E2 is the negative control plasmid without the HCV envelope protein sequence [59].

To study the effects of HVR1 swaps and the identified envelope protein substitutions on antibody binding, we expressed H77 and J4 variants of E1/E2 proteins (amino acids 192 to 747) in HEK 293T cells and extracted these using detergent. We performed ELISAs with lectin-coated plates to immobilize the E1/E2 extracted proteins and tested the binding of AR3A and AR4A. We found that binding of these antibodies was similar for H77-E1/E2 and H77_DH5-HVR1_-E1/E2 (Figs 9A and 9B and 9D and 9E, and S4A and S4B). In addition, binding was unaffected by the substitution I348N alone, while T385A conferred moderately increased AR4A binding to H77 (Figs 9B and S4B). In combination, the substitutions also moderately increased both AR3A and AR4A binding (Figs 9A and 9B and 9D and 9E, and S4A and S4B). Thus, our data demonstrate that extracted H77 and H77_DH5-HVR1_ E1/E2 protein complexes do not adequately recapitulate the properties of virion-associated E1/E2.

Performing similar ELISAs for J4-E1/E2, we observed low AR3A binding, which was greatly increased by the substitution F438V and increased further by combining F438V with F291I or V710L (Figs 9G and S4C), reflecting the neutralization sensitizing effect of these substitutions on J4 (Fig 8A). For J4_S52-HVR1_-E1/E2, the substitution F438V also increased AR3A binding (Figs 9J and S4C). However, the combination of F438V with V710L did not further increase AR3A binding to J4_S52-HVR1, F438V/V710L_-E1/E2, which contrasted the increased AR3A neutralization sensitivity of J4_S52-HVR1, F438V/V710L_ (Fig 8A). For AR4A, binding to both J4-E1/E2 and J4_S52-HVR1_-E1/E2 was increased by V710L as well as the combination of F291I/F438V (Figs 9H and 9K, and S4D), which was surprising given that we observed negligible effects of these substitutions on NAb sensitivity (Fig 8B). Overall, the data is in line with our conclusions from studying H77-E1/E2 antibody binding, suggesting that the effects of the described envelope protein substitutions on neutralization using virion-associated E1/E2 are moderately recapitulated on extracted E1/E2 protein of J4 for AR3A, while the effects for AR4A does not correlate between the two assays.

### Immunoprecipitation of HCVcc particles recapitulate the neutralization sensitivity phenotypes of the J4 recombinants but not the H77 recombinants

**Fig 10:**
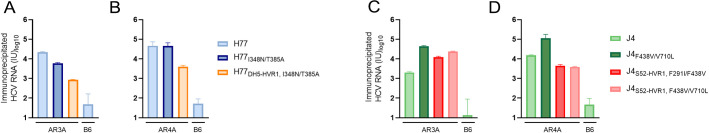
HVR1 swaps and associated adaptive envelope mutations have divergent effects on immunoprecipitation of HCVcc particles. Immunoprecipitation of H77 (A and B) and J4 (C and D) HCVcc recombinants with the indicated substitutions using AR3A and AR4A (B6 is a control for nonspecific binding). Magnetic beads were coated with the indicated mAbs and incubated with 10^6^ IU HCV RNA from infectious cell culture supernatant containing (A and B) H77, H77_I348N/T385A_ or H77_DH5-HVR1, I348N/T385A_ or (C and D) J4, J4_F438V/V710L_, J4_S52-HVR1, F291I/F438V_ or J4_S52-HVR1, F438V/V710L_. The amount of immunoprecipitated HCV RNA was measured by HCV RNA extraction of eluted fractions from the beads and reverse-transcription qPCR in duplicates as described in “HCV immunoprecipitation” in the Methods section. The data are shown as mean of technical duplicates with standard deviation (cut-off was 62 IU). The data shown is a representative experiment out of 2.

To also examine the effects of the identified substitutions on antibody binding of viral particles, we performed immunoprecipitation with AR3A and AR4A of H77 and J4 recombinants with different neutralization sensitivity phenotypes. For H77, we found that the substitutions I348N/T385A decreased immunoprecipitated HCV RNA by AR3A while having no effect on AR4A, whereas H77_DH5-HVR1, I348N/T385A_ had the lowest level of immunoprecipitated HCV RNA for both antibodies (Fig 10A and 10B). Thus, immunoprecipitation did not recapitulate the neutralization sensitizing effect of I348N/T385A in H77, nor did it reflect that H77_DH5-HVR1, I348N/T385A_ was equally neutralization sensitive as H77 (Fig 5). For J4, the substitutions F438V/V710L greatly increased HCV RNA immunoprecipitation by AR3A and to a lesser extent by AR4A (Fig 10C and 10D), thereby mimicking the effect of these substitutions on neutralization sensitivity (Fig 8). Furthermore, the level of AR3A immunoprecipitated HCV RNA was increased for J4_S52-HVR1, F291I/F438V_ and was even higher for J4_S52-HVR1, F438V/F710L_, while AR4A immunoprecipitated HCV RNA was lower for both recombinants, also mimicking the effect of these substitutions on neutralization sensitivity. Thus, our results suggest that differences in antibody binding explain the neutralization profiles of the J4 recombinants, but not the H77 recombinants.

### The HVR1-swap compensatory E1/E2 substitutions affect HCVcc entry dependency on SR-BI

**Fig 11:**
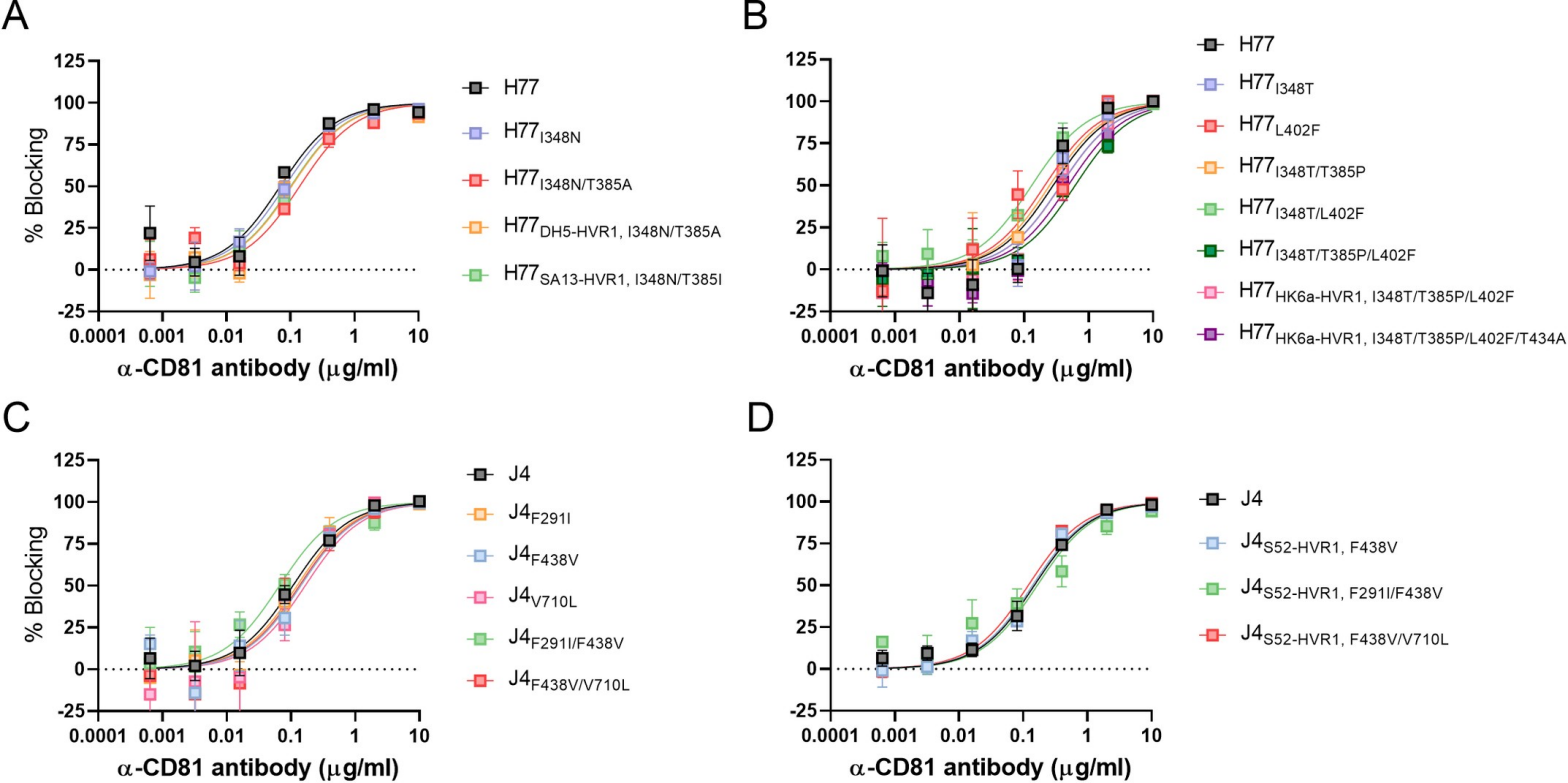
HVR1-swap compensatory E1/E2 substitutions do not affect CD81 entry dependency. HVR1-swap adaptive substitutions effect on H77 (A and B) or J4 (C and D) dependency on CD81. Huh7.5 cells were incubated with a 5-fold dilution series of anti-CD81 mAb JS-81 that specifically blocks the interaction between HCV and CD81 in four technical replicates with eight technical replicates of virus only and four technical replicates of control antibody. 48 hours post-infection the cells were immunostained for HCV antigen, and the number of FFUs/well was counted and normalized to the mean count of wells with virus only. Error bars represent standard deviation. The data were analysed using three-parameter sigmoid dose-response curves using Graphpad Prism v8.0.0 with bottom constraints set to 0.

**Fig 12:**
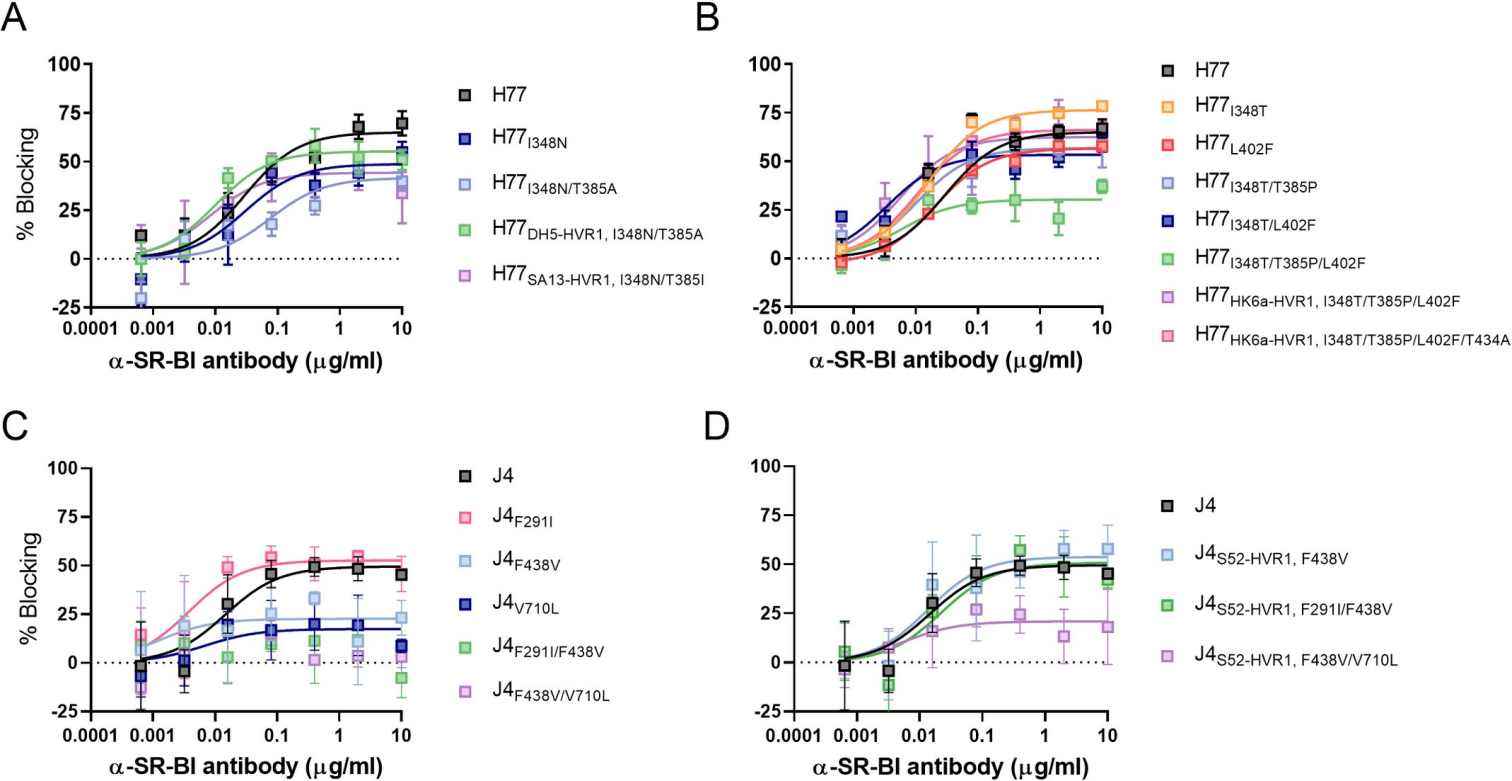
HVR1-swap compensatory E1/E2 substitutions typically decrease virus entry dependency on SR-BI. HVR1-swap adaptive substitutions effect on H77 (A and B) or J4 (C and D) dependency on SR-BI. Huh7.5 cells were incubated with a 5-fold dilution series of anti-SR-BI mAb, C16-71, which specifically blocks the interaction between HCV and SR-BI in four technical replicates with eight technical replicates of virus only and four technical replicates of control antibody D. 48 hours post-infection the cells were immunostained for HCV antigen, and the number of FFUs/well was counted and normalized to the mean count of wells with virus only. Error bars represent standard deviation. The data were analysed using three-parameter sigmoid dose-response curves using Graphpad Prism v8.0.0 with bottom constraints set to 0.

We have recently described polymorphic sites both within and outside HVR1 that alter HCV co-receptor dependency during entry [8]. Here, we wanted to study the influence of swapping HVR1 and the identified envelope substitutions on HCV entry dependency for co-receptors CD81 and SR-BI. Thus, we performed dose-response blocking assays using the generated 1^st^ passage virus stocks (S2 Table) with antibodies against CD81 and SR-BI. We found that all viruses were equally dependent on CD81 for entry (Fig 11), while SR-BI dependency appeared to be affected by some of the envelope substitutions (Fig 12). In the case of H77, SR-BI dependency was lowered by the substitution I348N and decreased further by the combination of substitutions I348N/T385A (Fig 12A). Also, the HVR1-swapped recombinants H77_DH5-HVR1, I348N/T385A_ and H77_SA13-HVR1, I348N/T385I_ were both less dependent on SR-BI for entry compared to H77 (Fig 12A). Furthermore, the substitution I348T, identified in the recovered H77_HK6a-HVR1_ recombinant, increased SR-BI dependency of H77, while the substitution T385P, also observed in the recovered H77_HK6a-HVR1_ recombinant, decreased the dependency of H77 on SR-BI (S5 Fig). The combinations of substitutions I348T/T385P, I348T/L402F and I348T/T385P/L402F decreased the dependency of H77 on this receptor (Fig 12B). However, H77_HK6a-HVR1, I348T/T385P/L402F_ and H77_HK6a-HVR1, I348T/T385P/L402F/T434A_ were both equally dependent on SR-BI as H77, suggesting that HK6a-HVR1 increase SR-BI dependency of H77 (Fig 12B).

For J4, SR-BI dependency was decreased by the substitutions F438V and V710L, and strikingly the introduction of the combinations F291I/F438V or F438V/V710L rendered the virus independent on SR-BI with no inhibition at the highest concentrations of anti-SR-BI antibody (Fig 12C). However, J4_S52-HVR1 F291I/F438V_ and J4_S52-HVR1, F438V/V710L_ were more dependent on SR-BI compared to J4 with these substitutions (Fig 12D), suggesting that SR-BI dependency of J4 was increased by S52-HVR1. Taken together, most compensatory substitutions found in the HVR1-swapped viruses decreased dependency on SR-BI for entry into host cells. Comparing the effect of these substitutions on the original recombinants with HVR1-swapped recombinants indicated that SR-BI dependency was also altered by the studied HVR1 swaps.

## Discussion

The roles of HVR1 in HCV NAb evasion and virus entry remain poorly understood. Here, we studied the function of HVR1 in cell entry and neutralization using a reverse genetics approach on attenuated HVR1-swapped HCVcc recombinants. We show that HVR1-swapped recombinants of HCV isolates H77 and J4 can recover infectivity in cell culture by acquiring compensatory envelope protein substitutions at otherwise conserved positions in E1 and E2. Moreover, these substitutions often increase the sensitivity of HCV to bNAbs targeting cross-genotype conserved epitopes while reducing HCV entry dependency on SR-BI. Importantly, the altered NAb sensitivity of HCV was not consistently recapitulated in binding experiments on detergent extracted E1/E2 and could only be reproduced with immunoprecipitated HCVcc particles of J4 recombinants, further highlighting the need for better experimental models to study these important phenomena at a molecular level. Increasing our understanding of HCV NAb evasion and the molecular changes of the envelope proteins could aid the efforts to design recombinant HCV antigens that will induce effective NAbs.

Due to high inherent flexibility, HVR1 is either partly or completely missing from solved E2 structures [14–17] and consequently, there is limited knowledge about the interaction of this protein region with the remaining part of the E1/E2 complex. We have previously shown that swapping HVR1 from other isolates into cell-culture infectious JFH1-based H77 recombinants has divergent effects on HCV infectivity [8], which we also describe here for HVR1-swapped J4 recombinants. To study HVR1-mediated NAb protection and to uncover clues on HVR1 interactions with the remaining part of the E1/E2 complex, we adapted highly attenuated HVR1-swap chimeras of H77 with HVR1 from DH5, SA13 or HK6a, as well as J4 with HVR1 from S52, to spread in cell culture. In all cases, we identified compensatory envelope protein substitutions that restored cell culture infectivity. For HVR1-swapped H77 recombinants, the substitutions mostly appeared at positions within HVR1 and in E1, whereas for HVR1-swapped J4 recombinants, substitutions appeared at other positions in E2, suggesting different pathways of adaptation. Comparing general E1E2 sequence homology (excluding HVR1) between H77 (genotype 1a) and isolates for which the HVR1 swap resulted in attenuation showed 82% amino acid similarity to DH5 (genotype 1b), 78% similarity to SA13 (genotype 5a) and 72% similarity to HK6a (genotype 6a). Interestingly, this same comparison of H77 with isolates for which HVR1 swap did not result in complete attenuation yielded homology ranging from 70% to 95% (TN (1a), DH1 (1b), J4 (1b), J6 (2a), S52 (3a) and ED43 (4a)) [8], indicating that HCVcc attenuation caused by some HVR1 swaps did not correlate with overall sequence similarity.

All the attenuated HVR1-swapped H77 recombinants and one out of five of the J4_S52-HVR1_ recombinants acquired compensatory substitutions within HVR1. Surprisingly, none of these substitutions reverted positions in HVR1 back to H77 or J4 sequence, respectively, but rather changed residues at otherwise highly conserved positions. Threonine at position 385, which is 99% conserved across genotype 1a and 96% conserved across all genotypes (Los Alamos HCV sequence database), was mutated in H77_DH5-HVR1_, H77_SA13-HVR1_, H77_HK6a-HVR1_ and in one case also in J4_S52-HVR1_. T385A was found to be cell culture adaptive for the SA13 JFH1-based Core-NS2 recombinant [28]. However, we found that this substitution decreased the infectivity of H77, showing that the adaptive effect is not universal. Furthermore, we found that substitutions in culture adapted H77_DH5-HVR1_, H77_SA13-HVR1_, and H77_HK6a-HVR1_ consistently appeared at position 348 in E1, which is 96% conserved across genotype 1a and 49% conserved across all genotypes (Los Alamos HCV sequence database). Interestingly, the amino acid changes did not introduce residues found in DH5, SA13 or HK6a, nor are they commonly observed in isolates in genotype 1b, 5a or 6a. Substitutions at position 348 were observed to be cell culture adaptive for the original H77, and other studies of genotype 1a, 2a and 6a have found adaptive substitutions close to this position, including M345T (isolate H77, genotype 1a) [29], I345V (isolate J6, genotype 2a), A349D (isolate H77, genotype 1a) [30], I347L (isolate Jc1, genotype 2a) [12] and T349S (isolate HK6a, genotype 6a) [11]. However, the adaptive effect of I348N was minor for the original H77 HCVcc, compared to the major increase in infectivity it conferred to H77_DH5-HVR1_ and H77_SA13-HVR1_, indicating that it also had HVR1-swap specific compensatory effects. This was further corroborated by our finding that cell culture adaptation of H77 did not induce adaptive changes at position 348. For H77_HK6a-HVR1_, the effect of I348T seemed to depend on the presence of other substitutions in HVR1, specifically T385P and L402F.

To further examine residues of HVR1 involved in the interaction with the remaining part of the E1/E2 complex, we performed in-depth reverse-genetics studies of the attenuated HVR1-swapped recombinant H77_DH5-HVR1_. We observed that viability of H77_DH5-HVR1_ was restored by reverting N-terminal HVR1 residues at positions 386, 388 and 393, indicating that residues at these positions were responsible for a potentially defective HVR1-E1/E2 interaction of this attenuated HVR1-swapped recombinant. The proximity to position 385, for which HVR1 swap adaptive substitutions were frequently observed, suggests that these substitutions serve a direct role in ameliorating the attenuating effect of specific residues at these nearby positions. Interestingly, two of these positions are located within a part of HVR1 (position 385–388) that, in a recent *in silico* model for the ectodomain of the E1/E2 heterodimer, were suggested to interact via a β-strand parallel coupling to residues 413–417 in AS412 [31]. Thus, substitutions at these positions could be affecting such an interaction. Recently, we showed that polymorphisms at position 400–404 (and perhaps 405) in the C-terminal part of HVR1 mediate broad NAb protection against antibodies targeting non-HVR1 epitopes, possibly by stabilizing a neutralization resistant conformational state of the envelope proteins [8]. Furthermore, we found that polymorphisms in E2 outside of HVR1 were dependent on the presence of HVR1 to regulate broad protection, suggesting that this resistant state depends on an interplay between HVR1 and the remaining part of E1/E2. These polymorphic sites also regulate SR-BI entry dependency. Here, we observed that substitutions at conserved positions in the N-terminal part of HVR1 are required for restoring infectivity of attenuated HVR1-swapped recombinants, while also influencing NAb sensitivity. As these substitutions appear at conserved positions it is tempting to speculate that they restore infectivity by disrupting HVR1 function through a mechanism that is different from the modulation of HVR1 function observed by polymorphic sites at position 400–404. The substitutions also reduce SR-BI entry dependency, further strengthening the link with broad NAb sensitivity.

HVR1-swapped J4 recombinants acquired compensatory substitutions outside HVR1 at positions different from H77, indicating that adaptation patterns were isolate-specific. As for H77, all identified substitutions in J4_S52-HVR1_ were at greatly conserved positions within genotype 1b (more than 95%) and not found in S52 or in other genotype 3a isolates (except for 291I which is present in <1% of the isolates). J4_S52-HVR1_ acquired the compensatory substitutions V710L or V710F in the stem region of E2. Interestingly, HVR1-deleted J4 acquired the compensatory substitution V710L [11] and V710F restored the infectivity of a H77 recombinant with E2 from J4 [32]. Thus, it seems that removing HVR1, swapping of HVR1 or introducing E1 from another isolate into J4 can give rise to substitutions at position 710. This suggests that a defect or missing interplay of HVR1 with the remaining part of E2 can be compensated for by changes in the E2 stem region that also regulates E1-E2 interactions, possibly linking functionality of HVR1 to the overall conformation of the E1/E2 complex.

While HVR1 is believed to protect HCV from NAbs [11, 12, 33], the intra-complex HVR1 interactions that govern this mechanism remain largely obscure. Here, we show that the identified compensatory substitutions of attenuated HVR1-swapped recombinants influenced viral sensitivity to different bNAbs. Overall the substitutions increased the sensitivity of H77 to AR3A [25] and AR4A [26], possibly by shifting global E1/E2 conformations from “closed” (neutralization-resistant) to “open” (neutralization-sensitive) states [8]. Interestingly, we found the bNAb-sensitizing effect of several of the substitutions to be mutually co-dependent. One of the sensitizing substitutions, T385A, has previously been shown to increase the neutralization sensitivity of SA13 Core-NS2 recombinants to both AR3A and AR4A [28], and in H77 HCVpp, this substitution increased sensitivity to patient serum [34]. It has also been shown that T385A increases the binding of AR4A to HEK293T cell expressed H77-E1E2 in flow cytometry assays [35]. In some genotype 1 isolates, position 385 contains a putative O-linked glycosylation site [34, 36]. Thus, it is possible that 385 is glycosylated in H77, and the increased sensitivity caused by the threonine to alanine change is due to disruption of O-linked glycan-mediated shielding.

Interestingly, we found that the cell culture adapted HVR1-swapped recombinants, H77_DH5-HVR1, I348N/T385A_ and H77_SA13-HVR1, I348N/T385I_, were similarly sensitive as H77 to AR3A and AR4A. Similarly, the HVR1-swapped recombinant, H77_HK6a-HVR1, I348T/T385P/L402F/T434A_, was as sensitive as H77 to AR4A, but highly resistant to AR3A. This resistance might be due to the substitution T434A, which is within the AR3A epitope in the E2core crystal structure [15], and thus a change at this position might directly affect AR3A epitope stability.

While the compensatory substitutions identified in J4_S52-HVR1_ increased J4 sensitivity to the E2-specific antibody, AR3A, they only had a minor effect on sensitivity to AR4A and AR5A, targeting non-overlapping epitopes in the interface between E1 and E2 [26]. This was most apparent for F438V, and the epitope-specific antibody sensitivity might suggest that this substitution somehow promotes local AR3A epitope exposure, which is interesting given the overlap of this epitope with the CD81 binding site. Previously, El-Diwany et al. has shown that L438V in the context of genotype 1a increased sensitivity to both HC33.4 and AR4A through a mechanism involving SR-BI [37]. We observed no effect of F438V on J4 sensitivity to AR4A, indicating that the NAb sensitivity modulating effect of this residue is dependent on the subtype-specific E1/E2 sequence. Since position 438 is proximal to known AR3A contact residues [15], F438V likely increased AR3A sensitivity by directly increasing AR3A binding to its epitope. This is supported by our recent findings of AR3A-specific resistance substitutions at this position in AR3A escape studies [29]. These findings, together with the fact that J4 has a rare amino acid, phenylalanine, at position 438 (<0.5% conserved across all genotypes), might explain why J4 is not easily neutralized by AR3A [38]. Interestingly, residue 438 is also believed to be part of a hydrophobic helix motif (residues 437–442) that provides a structural framework for the positioning of residues F442 and Y443, critical for interactions with CD81 [39]. HVR1 from S52 was recently found to confer a high level of protection of conserved epitopes compared to HVR1s from other isolates [8]. The combination of S52-HVR1 in the J4 E1/E2 backbone could be driving the acquisition of mutations that increase local exposure of CD81-binding residues in J4_S52-HVR1_ to promote interactions with this essential co-receptor.

Despite the general NAb-sensitizing effects of the HVR1 swap adaptive substitutions, we did not observe high sensitivity of adapted HVR1-swapped recombinants harboring these substitutions. This might infer that the attenuating HVR1 swap itself had the opposite effect (i.e. causing NAb resistance), but it should be noted that we lack neutralization data for HVR1-swapped recombinants without compensatory substitutions, which would be the appropriate viruses for a direct comparison.

We studied the effects of swapping HVR1 and the compensatory envelope substitutions on antibody binding by performing ELISAs on detergent-extracted HCV E1/E2 H77 and J4 variants. We found that binding of J4 E1/E2 variants to AR3A reflected the observed neutralization sensitivity of the corresponding J4 recombinants. However, we observed that substitutions which had no significant effect on AR4A neutralization increased the binding of J4 E1/E2 to this NAb. Additionally, the observed effect of the identified substitutions on AR3A and AR4A binding to H77 E1/E2 contrasted the effects observed for these substitutions on neutralization sensitivity of the corresponding H77 recombinants. Thus, E1/E2 behavior on viral particles was only partly recapitulated on expressed E1/E2 protein. This might suggest that the effect of the identified substitutions on neutralization sensitivity is dependent on interactions within higher-order structures of E1/E2 heterodimers, only present on viral particles or that it depends on interactions with non-envelope moieties, like apolipoprotein E [40], not expressed in HEK293T cells in which E1/E2 protein was produced.

To further examine the observed discrepancies in the effects of the envelope substitutions on antibody binding to extracted E1/E2 and HCVcc neutralization, we performed AR3A and AR4A mediated immunoprecipitation of selected H77 and J4 HCVcc recombinants. Surprisingly, immunoprecipitation data only recapitulated the neutralization sensitivity phenotypes for J4 recombinants and not for H77 recombinants. This might indicate that HVR1-swapped H77 adaptation relies on a mechanism not solely related to NAb binding itself and highlights the potential differences in how H77 and J4 overcome attenuation caused by HVR1 swaps. Interestingly, antibody binding to variants of non-extracted, cell-bound E1/E2 [23] and soluble E2 [8] have also been found to only partly recapitulate observations from neutralization of HCVcc. Thus, studies of HCV antibody evasion are made difficult by a lack of relevant models for studying these phenomena at a molecular level, and the interpretation of data from the mentioned approaches should always be evaluated critically.

HCV depends on several cellular factors for entry, including SR-BI [20] and CD81 [19]. Here, we observed no effect of the identified substitutions on dependency on CD81 but found that most substitutions decreased SR-BI entry dependency, as observed by others in cell culture adaptation of HCV recombinants [41]. We recently showed that HVR1, envelope polymorphisms and N-linked glycans modulate CD81 binding and alter SR-BI dependency in a complex manner [8, 23]. We hypothesized that SR-BI aids NAb-resistant HCV engage CD81 by shifting global E1/E2 conformational states from “closed” to “open” [8, 23], which is supported by observations of similar dynamic equilibria for other flaviviruses [42, 43]. This hypothesis is also supported by another study that suggests a link between neutralization resistant isolates and direct binding of SR-BI to isolate-specific HVR1 sequences [44]. Recently, a mathematical model was developed, where it was suggested that HCV could interact with CD81 either directly or with the prior engagement of SR-BI [45]. Based on this, the identified substitutions observed to decrease SR-BI entry dependency might promote direct viral engagement of CD81 by shifting E1/E2 conformations toward “open” states. This fits well with another recent study hypothesizing that E1/E2 hyperactivity is associated with HVR1 conformational entropy and/or dynamics [46]. They suggest that HVR1 acts as a safety catch with autoinhibitory functions that suppress the activity of E1/E2 on free virions. They further hypothesize that SR-BI binds and stabilizes HVR1, whereby autoinhibition is relaxed and subsequent downstream HCV entry events can occur. Thus, it is proposed that HVR1 dynamics provide a potential molecular mechanism that links E1/E2 reactivity, SR-BI dependency, entry efficiency and NAb sensitivity [46].

In conclusion, our study shows that swapping HVR1 between different HCV isolates has divergent effects on infectivity, suggesting sequence-dependent interactions between HVR1 and the rest of the E1/E2 complex. Highly attenuated, HVR1-swapped viruses were adapted in cell culture and acquired substitutions at isolate-dependent, but conserved, positions in both E1 and E2. The identified compensatory substitutions increased H77 sensitivity to bNAbs AR3A and AR4A and J4 sensitivity to AR3A while having opposite or negligible effects on J4 AR4A sensitivity. These effects were only partly recapitulated in ELISAs of extracted E1/E2 protein and reproduced in AR3A and AR4A immunoprecipitation of HCV particles for J4, but not for H77. As expected, the substitutions did not affect CD81 entry dependency of HCV, but many decreased SR-BI entry dependencies. Our study emphasizes the importance of assaying envelope protein substitutions on infectious virus particles since other available model systems of antibody binding may not recapitulate native E1/E2 protein features. The study also contributes to the understanding of HVR1-mediated protection of HCV cross-genotype conserved bNAb epitopes, indicating a mechanism that involves a complex interplay between HVR1 and the remaining part of E1/E2.

## Methods

### Cell Cultures

Huh7.5 cells [47] provided by Charles Rice (The Rockefeller University, New York, USA) and HEK293T cells (ATCC cell lines) were cultured in Dulbecco’s modified Eagle’s medium (DMEM) (Gibco/Invitrogen) supplemented with 10% heat-inactivated and filtered fetal bovine serum (FBS), 100 U/mL penicillin and 100 μg/ml streptomycin (Gibco/Invitrogen) with 5% CO_2_ at 37°C. The cells were split every 2 to 3 days.

### Virus isolates

We used JFH1-based Core-NS2 HCV recombinants H77/JFH1 [48] and J4/JFH1 [27] for generating HCVcc. HVR1 sequences were from HCV isolates DH5 [49], SA13 [50], HK6a [27], J6 [51, 52] and S52 [53]. All recombinants are referred to by the isolate name in the Core-NS2 region, which includes E1 and E2. HVR1 sequences and coding mutations were introduced into the recombinants using standard molecular cloning with QuikChange Lightning Multi Site-Directed Mutagenesis kit (Agilent). The HCV sequence for each plasmid was confirmed by Sanger sequencing (Macrogen Europe) of the final DNA preparation.

### Antibodies and reagents

NS5A antibody, 9E10 [51], was kindly provided by Charles Rice (The Rockefeller University, New York, USA). The monoclonal antibodies (mAbs) b6 (negative control) and AR3A, AR4A and AR5A [25, 26], specific for HCV envelope proteins, were kindly provided by Mansun Law (The Scripps Research Institute, California, USA). For receptor blocking assays, we used anti-CD81 mAb JS-81 (BD Pharmingen, Cat No. JS81) and anti-SR-BI mAb C16-71 [30, 54]. Control antibodies for receptor blocking assays were isotype antibody 553447 for CD81 (BD Pharmingen) and antibody D for SR-BI [54]. For western blotting, we used AP33 [55] kindly provided by Arvind Patel (University of Glasgow, Glasgow, Scotland).

### Transfection of Huh7.5 cells

HCV transfections were performed as described [30]. Briefly, the plasmids were linearized with XbaI (New England Biolabs) and used to generate HCV RNA transcripts that were transfected into Huh7.5 cells using Lipofectamine 2000 (Invitrogen). For 24-96-hour transfections, viral spread and release of infectious particles were monitored every 24 hours by HCV-specific immunostaining of transfected cells and analysis of HCV infectivity titers from transfection supernatants collected from individual cultures. For long-term transfections, the cultures were evaluated every 2 or 3 days by HCV-specific immunostaining. Supernatant was collected when the virus had spread to ~80% of the cells. Percentage spread was determined by immunostaining using anti-NS5A antibody (9E10) and secondary Alexa Flour 488 goat anti-mouse IgG (Invitrogen), and Hoechst 33342 dye for fluorescent staining of cell nuclei. The sequences of the envelope E1 and E2 or the entire ORF of culture derived HCV RNA were determined as described [11, 56] and analyzed using Sequencher 5.3 (Gene Codes Corporation). The infectivity titers were determined in triplicate by plating 7 × 10^3^ cells/well on poly-D-lysine-coated 96-well plates (Nunc). The next day different supernatant dilutions containing the viral particles were added to the Huh7.5 cells on the 96-well plates for a 48 hours incubation at 37°C with 5% CO_2_. The cells were fixed with methanol and analyzed using HCV-specific NS5A immunostaining. The number of FFUs was determined as described [53, 56].

### Generation of 1^st^ passage virus stocks

2 × 10^5^ Huh7.5 cells/well were plated in 6-well plates and incubated for 24 hours at 37°C with 5% CO_2_. Cells were inoculated with 300–1000 μl supernatant collected from the 24-96-hour transfections. 24 hours after inoculation, the cells were transferred to T25 flasks and subsequently into T80 flasks after another 2–3 days. When the virus had spread to ~25% of the cells (determined by immunostaining), the cells were transferred to T175 flasks. At two timepoints around the peak of infection, supernatant was collected from the T175 flasks. Virus stock infectivity titers were determined, and the sequences of the envelope E1 and E2 or the entire ORF of culture derived HCV RNA were determined as described [11, 56] and analyzed using Sequencher 5.3 (Gene Codes Corporation) to confirm virus sequence.

### Antibody neutralization assay

Cells were plated at 7 × 10^3^ Huh7.5 cells/well in poly-D-lysine 96-well plates and incubated for 24 hours. The following day mAbs were diluted in DMEM (supplemented with 10% FBS and antibiotics) in a 5-fold dilution series. The mAb dilutions were mixed with virus stocks corresponding to readouts of >10 to <200 FFU/well and together with 8 technical replicates of virus only incubated in a 37°C and 5% CO_2_ incubator for 1 hour and then added to Huh7.5 cells for a 4-hour infection under similar conditions. A relevant control antibody (b6) was included for each virus. After the 4-hour infection, the cells were washed with prewarmed PBS, and fresh medium was added before a final incubation for 48 hours (total infection time) at 37°C and 5% CO_2_. Cells were fixed and immunostained for NS5A, as previously described. The number of FFUs was counted using an ImmunoSpot Series 5 UV Analyzer as described [53, 56]. The data were normalized to 8 technical replicates of virus only and analyzed using three-parameter curve fitting in GraphPad Prism 8 with bottom constraint set to 0 and top set to 100.

### Receptor blocking assays

7 × 10^3^ Huh7.5 cells/well were plated on poly-D-lysine 96-well plates and incubated for 24 hours. A 5-fold dilution series of mAbs against CD81 or SR-BI and a single high dose of the respective control antibodies were prepared in four technical replicates and added to the cells for 1-hour incubation at 37°C and 5% CO_2_. Following this, virus stock corresponding to a readout of >50 to <200 FFU/well was added to the cell/antibody mix. After 4-hour infection, the cells were washed with prewarmed PBS, and fresh medium was added before a final incubation for 48 hours (total infection time) at 37°C and 5% CO_2_. The cells were fixed, immunostained with NS5A antibody (9E10), and the number of FFUs was counted as described [53, 56]. The data were normalized to 8 technical replicates of virus only and analyzed using three-parameter curve fitting in GraphPad Prism 8, bottom set to 0.

### ELISA for measuring antibody binding to extracted E1/E2 protein

mAb binding to E1/E2 was quantified using an ELISA as previously described [57]. Briefly, HEK293T cells were transfected with E1/E2 expression constructs [32, 58]. After 48 hours, cell lysates were harvested with extraction buffer containing 0.5% DDM. Samples were treated with benzonase (Sigma Cat No. E1014) for 1 hour at room temperature. 96-well plates were coated with 5 μg/ml of *Galanthus nivalis* lectin (Sigma Cat No. L8275) at 4°C overnight. The plates were blocked with PBS containing 0.1% Tween-20 (Sigma) and 5% non-fat dry milk. The E1/E2-containing cell lysates were added to the plates and incubated overnight at 4°C. mAb binding was analyzed in duplicate in a 5-fold dilution series, starting at 50 μg/ml. Binding was detected with an HRP-conjugated anti-human IgG secondary antibody (Invitrogen, Cat No. A24476). Stabilized chromogen substrate (Biosource, Cat No. S802) was added, and incubated for 15 min and the reaction was stopped with sulfuric acid. Absorbance was measured at 450 nm.

### SDS-page and western blotting for estimation of E1/E2 protein levels

The cell lysates prepared in DDM were analyzed for E2 content. NuPAGE LDS sample buffer (4x) (Thermo Fisher Scientific) and NuPAGE Sample Reducing Agent (10X) (Thermo Fisher Scientific) were added to the samples and heated for 10 min at 70°C for denaturing electrophoresis. The samples together with a protein molecular marker (Bio-Rad, Cat No. 1610376) were loaded on a NuPAGE Novex 10% Bis-Tris gel (Invitrogen) and run in an XCell II SureLock Mini-Cell (Invitrogen). The proteins were transferred to a polyvinylidene difluoride (PVDF) membrane using electroblotting with 1x NuPAGE transfer buffer (Invitrogen) + 10% ethanol. Membranes were blocked with BSK and stained with anti-E2 primary mAb, AP33 [55] and anti-beta actin antibody (ab8227, abcam) at 4°C overnight. The protein bands were visualized with Alexa Flour 488 goat anti-mouse IgG (Invitrogen) and Alexa Flour 647 goat anti-rabbit IgG (Invitrogen) for 1 hour at room temperature and detected using Image Lab 5.2.1, Bio-Rad.

### HCV immunoprecipitation

Dynabeads magnetic beads (50 μl; Immunoprecipitation Kit, Dynabeads Protein G, Invitrogen cat. no. 10007D) were resuspended in 200 μl antibody binding and washing buffer and incubated with 5 μg of antibody (AR3A, AR4A or b6) on a shaker for 30 min at room temperature. Subsequently, the beads were washed twice and incubated with the relevant virus (10^6^ international units [IU] HCV RNA) in 500 μl complete medium on a shaker for 1 hour at room temperature. Afterwards, the beads were washed five times in 200 μl washing buffer before the RNA was eluted in 22 μl elution buffer. The beads were removed by flicking the sample for 30 sec. Duplicates of 10 μl of the eluate were used for RNA extraction (RNA clean & Concentrator-5, Zymo Research, cat. no. R1016) together with a dilution series of controls with known HCV RNA concentrations of 0–5 x 10^6^ IU/ml in 1-log increments. The extracted RNA was used for reverse-transcription quantitative PCR as described in [53] using a 7500 real-time PCR System (Applied Biosystems). HCV RNA titers (IU/ml) were calculated using the standard curve from the controls and the corresponding cycle threshold (Ct) value. The detection limit was 500 IU/ml.

## Supporting information

S1 Table Coding mutations observed in the HCV envelope protein sequences for H77 HCVcc recombinants with HVR1 from DH5, SA13 or HK6a. Sequence analysis of amplified nucleotide envelope protein sequences from cell culture adapted HVR1-swapped recombinants. HCV positive cells were passaged for the indicated number of days until the virus had spread to >80% of the cells. Only coding mutations are included. At positions with nucleotide mixtures; lower-case letters denote the minor sequence; upper-case letters denote the most prevalent sequence. Two letters written with upper-case letters denote two nucleotides present in comparably equal amounts. Dots indicate the original plasmid sequence. Amino acid positions are numbered according to H77 abs. ref. (GenBank #AF009606).
(TIF)

S2 Table Titrated stocks of H77 and J4 HCVcc recombinants. HCV envelope gene sequences, including the presence of specific substitutions, were verified by direct sequencing of culture-derived HCV RNA. HCV infectivity titers are given in log10 FFU/ml per ml and represent a mean of three technical replicates. The substitutions are numbered according to H77 abs. ref. (GenBank #AF009606).
(TIF)

S3 Table Coding mutations observed in the HCV envelope proteins sequences for J4_S52-HVR1_ HCVcc recombinants. Sequence analysis of amplified envelope protein sequences from cell culture adapted HVR1-swapped HCVcc recombinants. HCV-positive cells were passaged for the indicated number of days until the virus had spread to >80% of the cells. Only coding mutations are included. At positions with nucleotide mixtures; lower-case letters denote the minor sequence; upper-case letters denote the most prevalent sequence. Two letters written with upper-case letters denote two nucleotides present in comparably equal amounts. Dots indicate the original plasmid sequence. Amino acid positions are numbered according to H77 abs. ref. (GenBank #AF009606).
(TIF)

S4 Table Clonal analysis of envelope protein sequences for cell culture adapted J4_S52-HVR1_ HCVcc recombinant from Experiment 5. Clonal sequence analysis of the envelope genes of the adapted J4_S52-HVR1_ HCVcc (S3 Table exp. 5). Coding mutations were identified by TOPO XL cloning of the RT-PCR product of the adapted recombinant. Dots indicate the original plasmid sequence. The mutations are numbered according to H77 abs. ref. (GenBank #AF009606).
(TIF)

S5 Table Coding nucleotide changes in entire ORF of adapted J4 and J4_S52-HVR1_ HCVcc recombinants. Full ORF sequence analysis of RT-PCR of J4 and J4_S52-HVR1_ adapted in Huh7.5 cells. HCV-positive cell cultures were passaged for the indicated number of days until the virus had spread to >80% of the cells. Supernatant was collected at the peak of infection and used for sequence analysis of the full ORF. Only coding mutations are included. At positions with nucleotide mixtures; lower-case letters denote the minor sequence; upper-case letters denote the most prevalent sequence. Two letters written with upper-case letters denote two nucleotides present in comparably equal amounts. Dots indicate the original plasmid sequence. Amino acid positions are numbered according to H77 abs. ref. (GenBank #AF009606). Blue cells show cell culture adaptive mutations identified by *Gottwein et al*., *2009* [27].
(TIF)

S1 Fig Infectivity of H77_DH5-HVR1_ is restored by introducing the H77 residue at the N-terminal HVR1 positions 386, 388 and 393. Huh7.5 cells were transfected with *in vitro* transcribed HCV RNA of the indicated recombinants (A–C). Supernatants were collected 24, 48, 72 and 96 hours post transfection, and HCV infectivity titers were determined. At each timepoint, infectivity titers are represented by a mean of three technical replicates. Error bars show standard deviation. Lower level of quantification was 100 FFU/ml. The substitutions are numbered according to H77 abs. ref. (GenBank #AF009606). The data shown is a representative experiment out of at least 2.
(TIF)

S2 Fig J4 HCVcc recombinant with S52-HVR1 does not produce infectious particles in Huh7.5 cells. Huh7.5 cells were transfected with *in vitro* transcribed HCV RNA of the indicated recombinants. Supernatants were collected 24, 48, 72 and 96 hours post transfection, and HCV infectivity titers were determined. At each timepoint, infectivity titers are represented by a mean of three technical replicates. Error bars show standard deviation. Lower level of quantification was 100 FFU/ml.
(TIF)

S3 Fig E1/E2 substitutions identified in HVR1-swapped J4 recombinant have little effect on sensitivity of J4 recombinants to AR5A. Neutralization by the bNAb AR5A of the indicated recombinants. The viruses were incubated in four technical replicates with a 5-fold dilution series of AR5A, starting at 50 μg/ml, along with eight technical replicates of virus only. 48 hours post-infection the cells were immunostained for HCV antigen, and the number of FFUs/well was counted and normalized to the mean count of wells with virus only. Each dot represents the mean IC_50_ value of the indicated recombinants. The broken line represents the IC_50_ value of unmodified J4. Error bars represent the 95% confidence intervals (IC_95_). The data was analyzed using three-parameter dose-response with the top value set to 100 and lower value set to 0, to calculate IC_50_ and IC_95_ using GraphPad Prism v8.0.0.
(TIF)

S4 Fig Half-maximal binding concentration values for AR3A or AR4A binding to extracted E1/E2 protein in ELISA. Half-maximal binding concentration values for AR3A or AR4A binding to extracted E1/E2 protein, H77 (A and B) or J4 (C and D). Cell lysates containing E1/E2 protein were added to lectin-coated ELISA plates and incubated overnight. mAbs were added in two technical replicates in a 5-fold dilution series for a 2-hour incubation. The binding was detected with an HRP-conjugated anti-human IgG secondary antibody. TMB Stabilized Chromogen substrate (Thermo Fisher Scientific) was added and the reaction was stopped using HCl prior to absorbance measurement at 450 nm. Each dot represents the mean OD_50_ value of the indicated proteins. The broken line represents the OD_50_ value of unmodified H77, H77_DH5-HVR1_, J4 or J4_S52-HVR1_. The data was analyzed using three-parameter nonlinear regression using Graphpad Prism v8.0.0. The data shown is a representative experiment out of at least 2.
(TIF)

S5 Fig I348T increases H77 entry dependency on SR-BI, while T385P decreases H77 entry dependency on SR-BI. Huh7.5 cells were incubated with a 5-fold dilution series of anti-SR-BI mAb, C16-71, which specifically blocks the interaction between HCV and SR-BI in four technical replicates with eight technical replicates of virus only and four technical replicates of control antibody D. 48 hours post-infection the cells were immunostained for HCV antigen, and the number of FFUs/well was counted and normalized to the mean count of wells with virus only. Error bars represent standard deviation. The data were analysed using three-parameter sigmoid dose-response curves using Graphpad Prism v8.0.0 with bottom constraints set to 0.
(TIF)
