# Supplementary figures and images for: In vitro adaptation and characterization of attenuated hypervariable region 1 swap chimeras of hepatitis C virus
Source: PLoS Pathog. 2021 Jul 19;17(7):e1009720. doi: 10.1371/journal.ppat.1009720 (PMC8321405; doi:10.1371/journal.ppat.1009720)

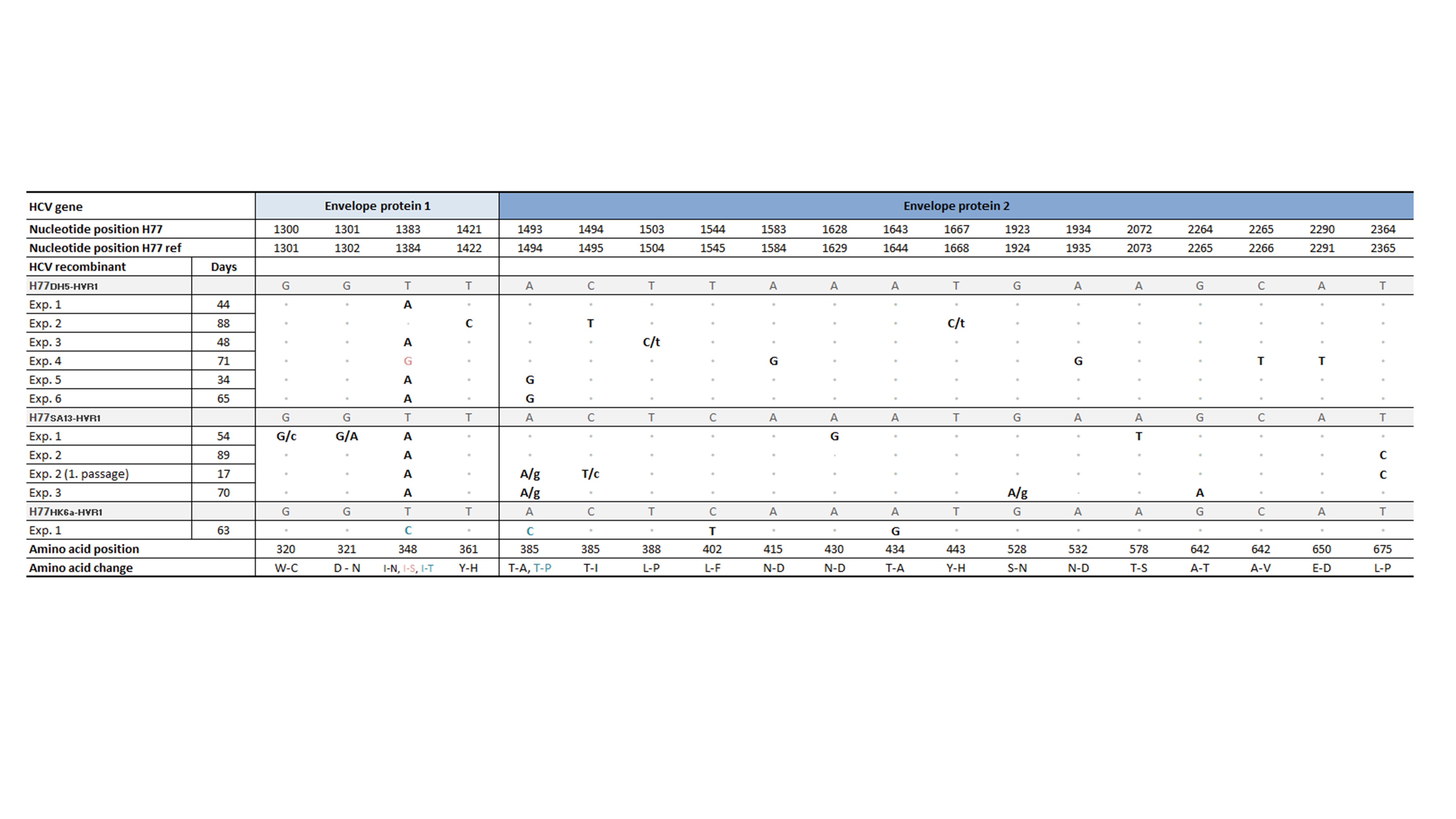

Supplement: S1 Table — Sequence analysis of amplified nucleotide envelope protein sequences from cell culture adapted HVR1-swapped recombinants. HCV positive cells were passaged for the indicated number of days until the virus had spread to >80% of the cells. Only coding mutations are included. At positions with nucleotide mixtures; lower-case letters denote the minor sequence; upper-case letters denote the most prevalent sequence. Two letters written with upper-case letters denote two nucleotides present in comparably equal amounts. Dots indicate the original plasmid sequence. Amino acid positions are numbered according to H77 abs. ref. (GenBank #AF009606). (TIF) [file ppat.1009720.s001.tif]

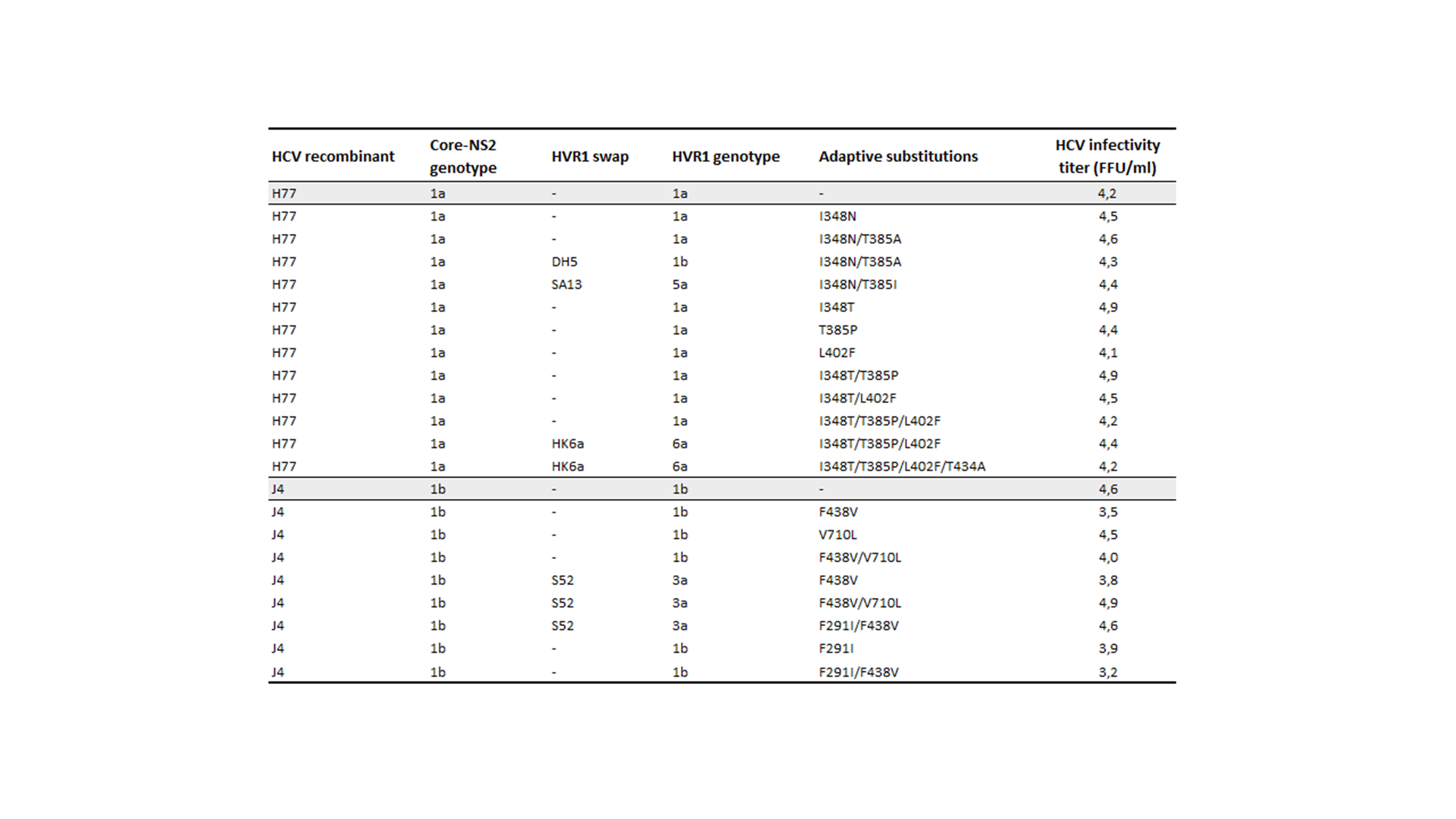

Supplement: S2 Table — HCV envelope gene sequences, including the presence of specific substitutions, were verified by direct sequencing of culture-derived HCV RNA. HCV infectivity titers are given in log10 FFU/ml per ml and represent a mean of three technical replicates. The substitutions are numbered according to H77 abs. ref. (GenBank #AF009606). (TIF) [file ppat.1009720.s002.tif]

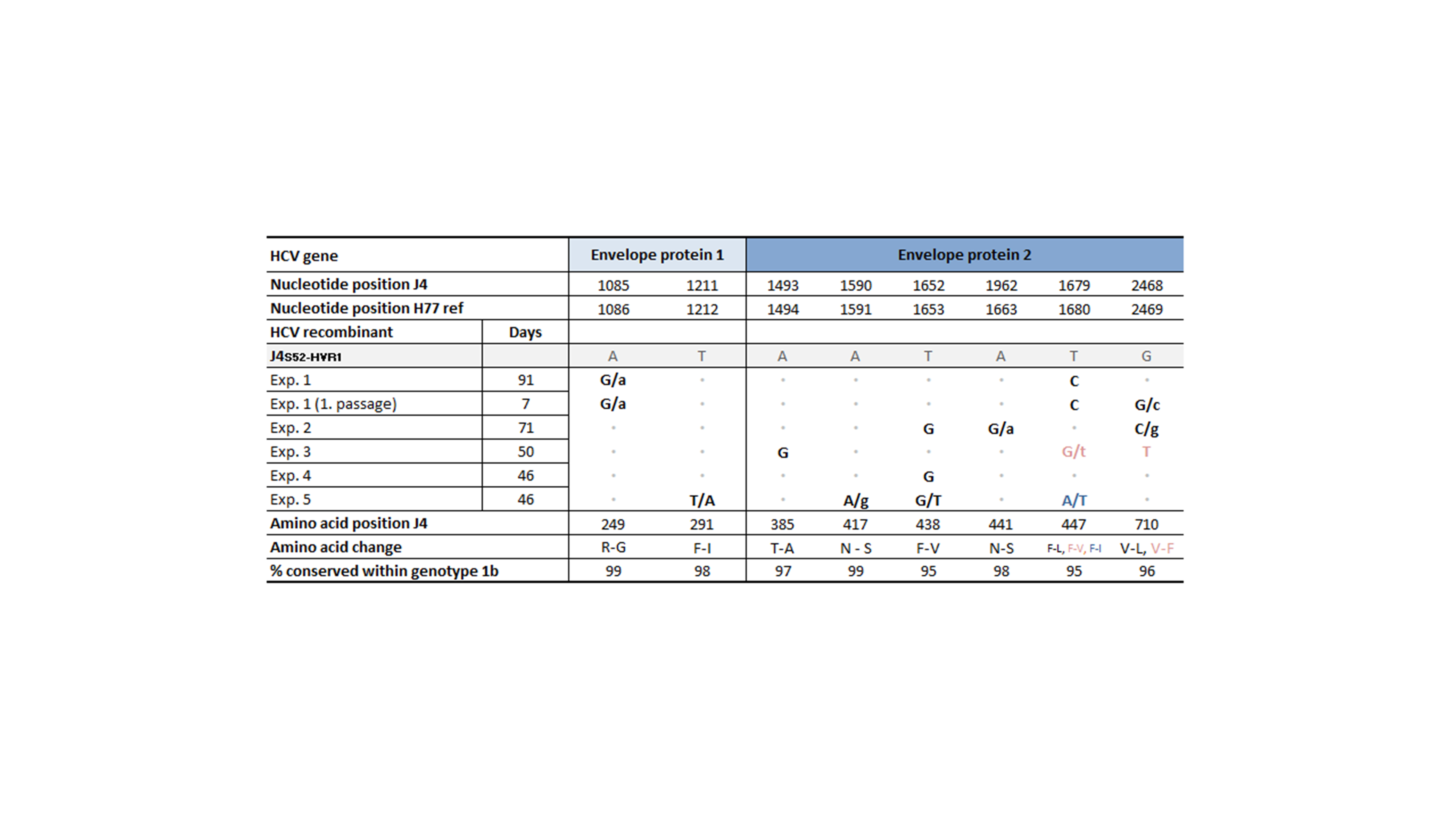

Supplement: S3 Table — Sequence analysis of amplified envelope protein sequences from cell culture adapted HVR1-swapped HCVcc recombinants. HCV-positive cells were passaged for the indicated number of days until the virus had spread to >80% of the cells. Only coding mutations are included. At positions with nucleotide mixtures; lower-case letters denote the minor sequence; upper-case letters denote the most prevalent sequence. Two letters written with upper-case letters denote two nucleotides present in comparably equal amounts. Dots indicate the original plasmid sequence. Amino acid positions are numbered according to H77 abs. ref. (GenBank #AF009606). (TIF) [file ppat.1009720.s003.tif]

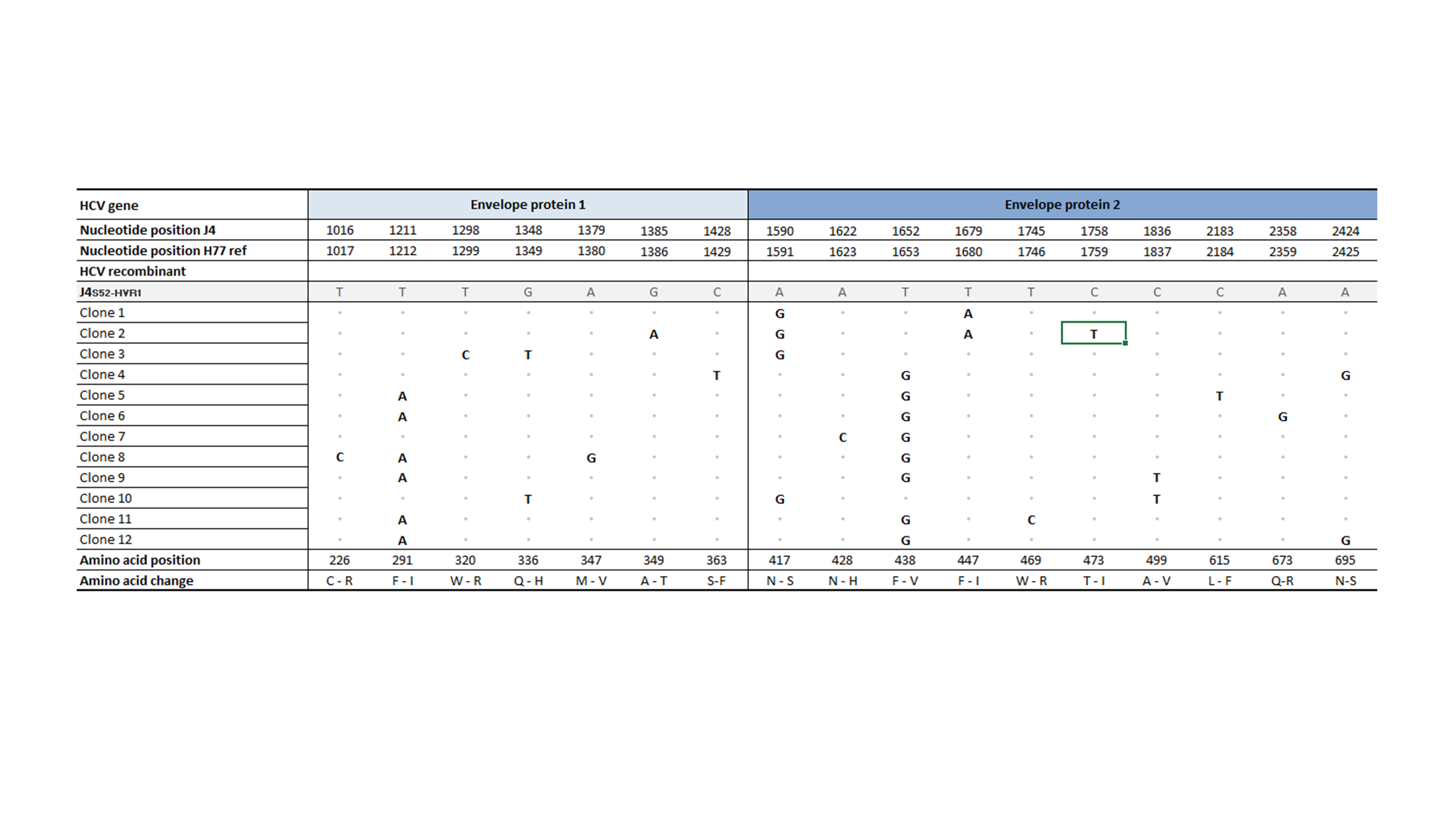

Supplement: S4 Table — Clonal sequence analysis of the envelope genes of the adapted J4S52-HVR1 HCVcc (S3 Table exp. 5). Coding mutations were identified by TOPO XL cloning of the RT-PCR product of the adapted recombinant. Dots indicate the original plasmid sequence. The mutations are numbered according to H77 abs. ref. (GenBank #AF009606). (TIF) [file ppat.1009720.s004.tif]

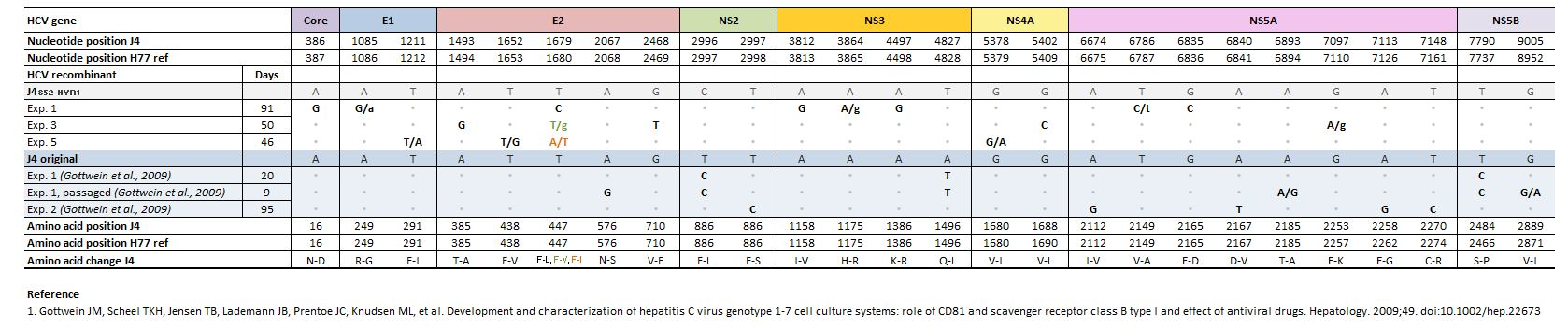

Supplement: S5 Table — Full ORF sequence analysis of RT-PCR of J4 and J4S52-HVR1 adapted in Huh7.5 cells. HCV-positive cell cultures were passaged for the indicated number of days until the virus had spread to >80% of the cells. Supernatant was collected at the peak of infection and used for sequence analysis of the full ORF. Only coding mutations are included. At positions with nucleotide mixtures; lower-case letters denote the minor sequence; upper-case letters denote the most prevalent sequence. Two letters written with upper-case letters denote two nucleotides present in comparably equal amounts. Dots indicate the original plasmid sequence. Amino acid positions are numbered according to H77 abs. ref. (GenBank #AF009606). Blue cells show cell culture adaptive mutations identified by Gottwein et al., 2009 [27]. (TIF) [file ppat.1009720.s005.tif]

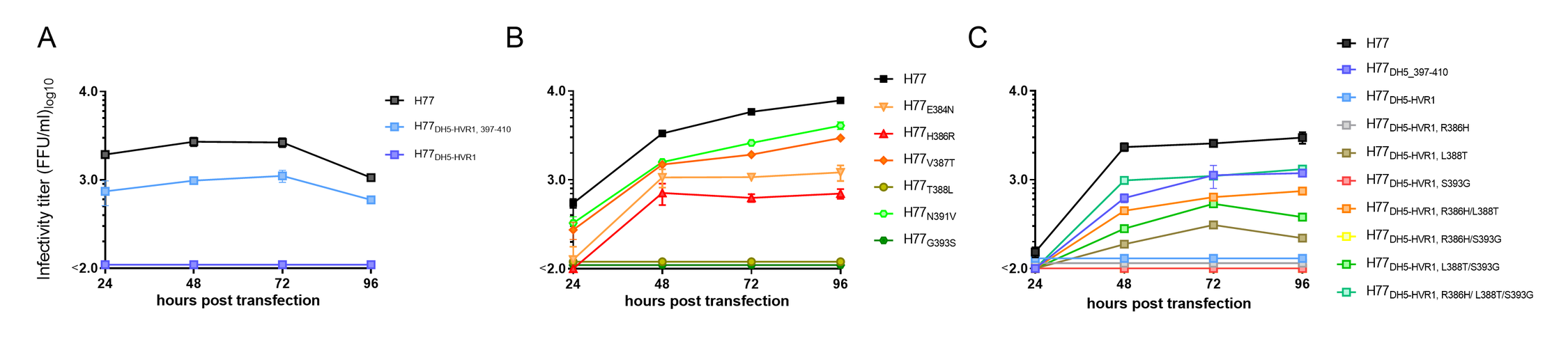

Supplement: S1 Fig — Huh7.5 cells were transfected with in vitro transcribed HCV RNA of the indicated recombinants (A–C). Supernatants were collected 24, 48, 72 and 96 hours post transfection, and HCV infectivity titers were determined. At each timepoint, infectivity titers are represented by a mean of three technical replicates. Error bars show standard deviation. Lower level of quantification was 100 FFU/ml. The substitutions are numbered according to H77 abs. ref. (GenBank #AF009606). The data shown is a representative experiment out of at least 2. (TIF) [file ppat.1009720.s006.tif]

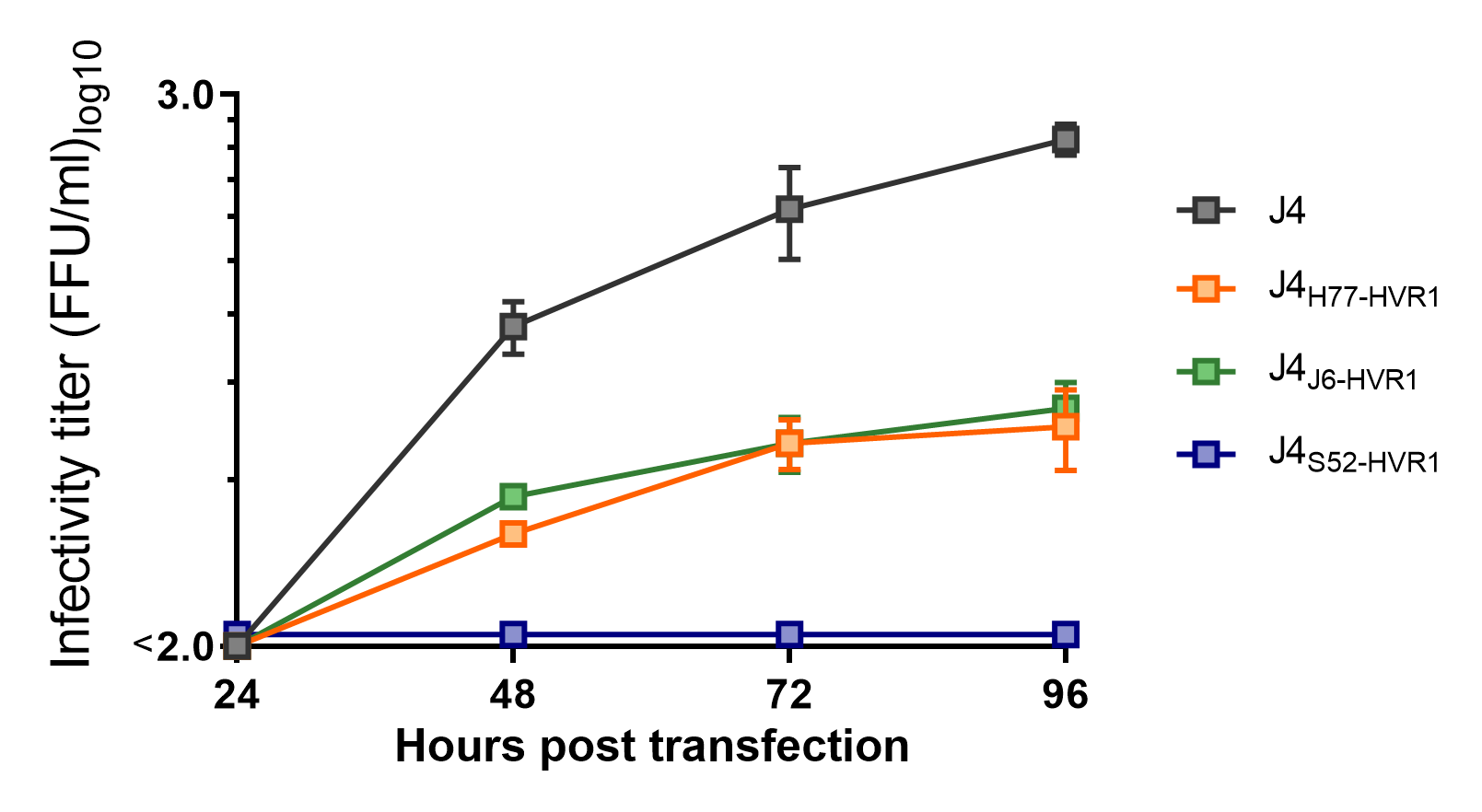

Supplement: S2 Fig — Huh7.5 cells were transfected with in vitro transcribed HCV RNA of the indicated recombinants. Supernatants were collected 24, 48, 72 and 96 hours post transfection, and HCV infectivity titers were determined. At each timepoint, infectivity titers are represented by a mean of three technical replicates. Error bars show standard deviation. Lower level of quantification was 100 FFU/ml. (TIF) [file ppat.1009720.s007.tif]

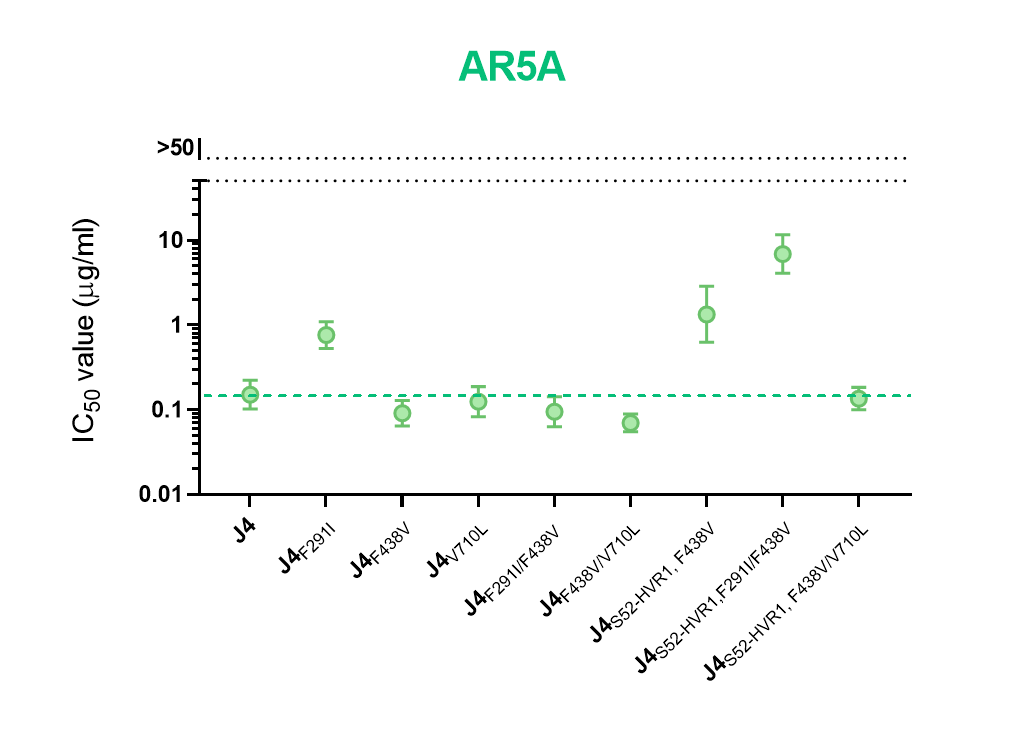

Supplement: S3 Fig — Neutralization by the bNAb AR5A of the indicated recombinants. The viruses were incubated in four technical replicates with a 5-fold dilution series of AR5A, starting at 50 μg/ml, along with eight technical replicates of virus only. 48 hours post-infection the cells were immunostained for HCV antigen, and the number of FFUs/well was counted and normalized to the mean count of wells with virus only. Each dot represents the mean IC50 value of the indicated recombinants. The broken line represents the IC50 value of unmodified J4. Error bars represent the 95% confidence intervals (IC95). The data was analyzed using three-parameter dose-response with the top value set to 100 and lower value set to 0, to calculate IC50 and IC95 using GraphPad Prism v8.0.0. (TIF) [file ppat.1009720.s008.tif]

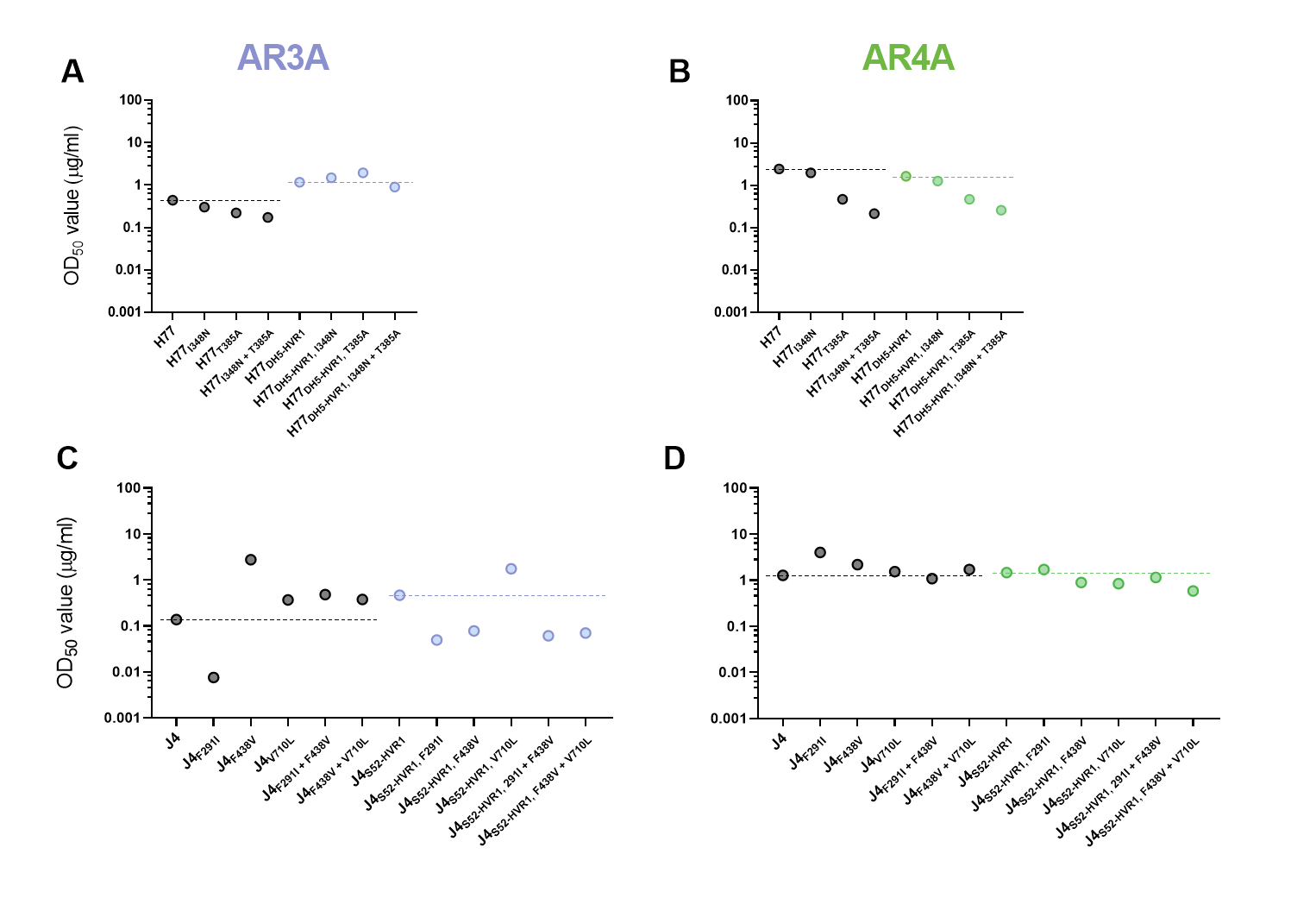

Supplement: S4 Fig — Half-maximal binding concentration values for AR3A or AR4A binding to extracted E1/E2 protein, H77 (A and B) or J4 (C and D). Cell lysates containing E1/E2 protein were added to lectin-coated ELISA plates and incubated overnight. mAbs were added in two technical replicates in a 5-fold dilution series for a 2-hour incubation. The binding was detected with an HRP-conjugated anti-human IgG secondary antibody. TMB Stabilized Chromogen substrate (Thermo Fisher Scientific) was added and the reaction was stopped using HCl prior to absorbance measurement at 450 nm. Each dot represents the mean OD50 value of the indicated proteins. The broken line represents the OD50 value of unmodified H77, H77DH5-HVR1, J4 or J4S52-HVR1. The data was analyzed using three-parameter nonlinear regression using Graphpad Prism v8.0.0. The data shown is a representative experiment out of at least 2. (TIF) [file ppat.1009720.s009.tif]

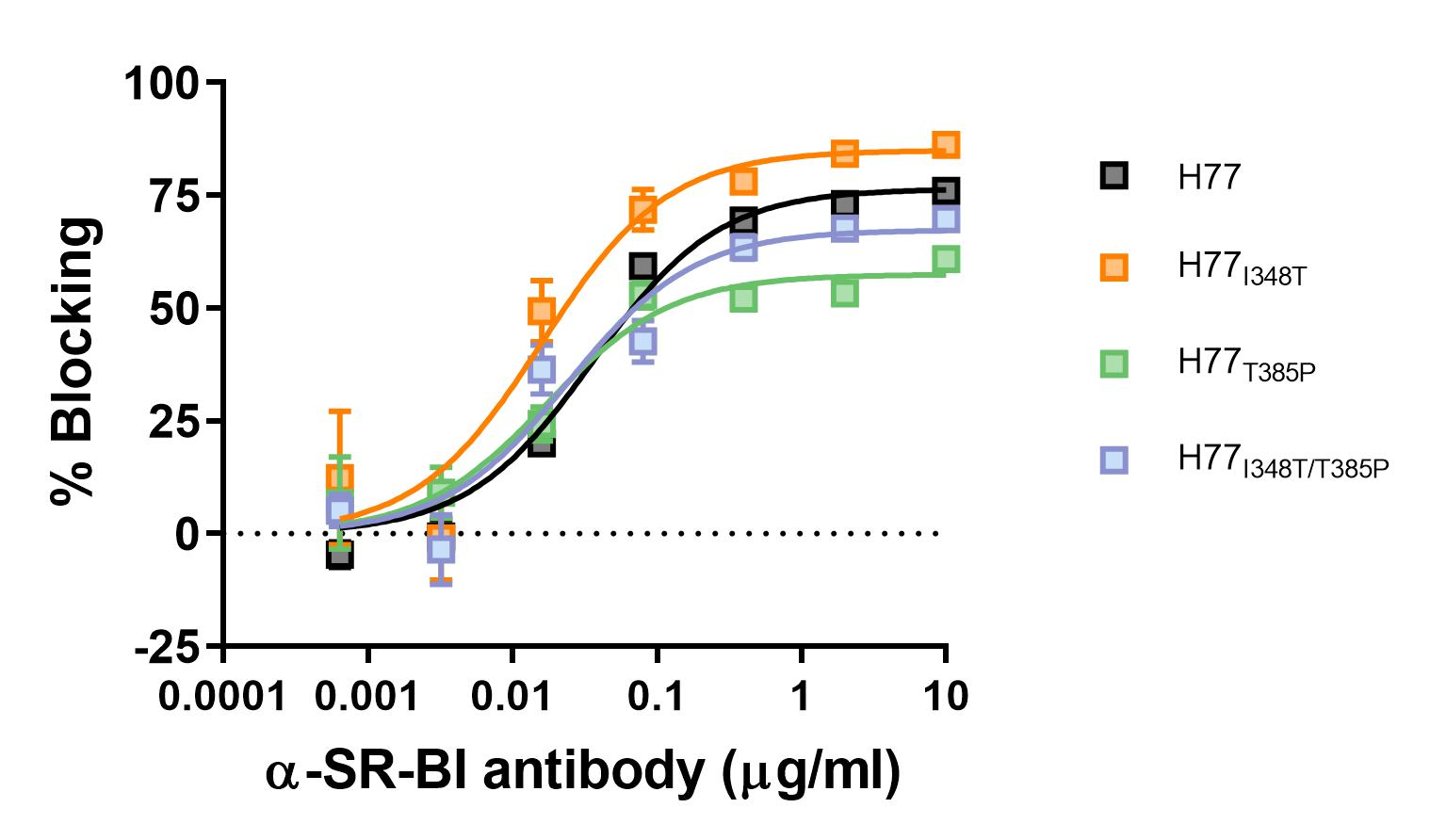

Supplement: S5 Fig — Huh7.5 cells were incubated with a 5-fold dilution series of anti-SR-BI mAb, C16-71, which specifically blocks the interaction between HCV and SR-BI in four technical replicates with eight technical replicates of virus only and four technical replicates of control antibody D. 48 hours post-infection the cells were immunostained for HCV antigen, and the number of FFUs/well was counted and normalized to the mean count of wells with virus only. Error bars represent standard deviation. The data were analysed using three-parameter sigmoid dose-response curves using Graphpad Prism v8.0.0 with bottom constraints set to 0. (TIF) [file ppat.1009720.s010.tif]
